# The Legacy of Mercury Contamination from Colonial Nonferrous Mining in the Southern Hemisphere

**DOI:** 10.1021/acs.est.5c03607

**Published:** 2025-06-17

**Authors:** Larissa Schneider, Saul Guerrero, Gavin Mudd, Marco A. A. Lopez, Kristen K. Beck, Ruoyu Sun, Simon G. Haberle, Michael-Shawn Fletcher, Atun Zawadzki, Holger Hintelmann, Alan Griffiths, Colin Cooke, Patrice de Caritat

**Affiliations:** † Department of Archaeology and Natural History, School of Culture, History and Language, 2219 The Australian National University, Coombs Bld. 9, Fellows Rd, Canberra, Australian Capital Territory 0200, Australia; ‡ Australian Research Council Centre of Excellence for Indigenous and Environmental Histories and Futures, The Australian National University, Canberra, Australian Capital Territory 2601, Australia; § Critical Minerals Intelligence Centre, 41819 British Geological Survey, Keyworth, Nottingham NG12 5GG, United Kingdom; ∥ 42573 Centro de Investigación en Matemáticas (CIMAT), Jalisco s/n, Valenciana, 36023 Guanajuato, Guanajuato, Mexico; ⊥ Catchments and Coasts Research Group, Department of Geography, 4547 University of Lincoln, Brayford Pool Campus, Lincoln LN6 7TS, United Kingdom; # Institute of Surface-Earth System Science, School of Earth System Science, Tianjin University, Tianjin 300072, China; g School of Geography, Earth and Atmospheric Sciences, The University of Melbourne, 3010 Carlton, Victoria, Australia; h Australian Research Council Centre of Excellence for Indigenous and Environmental Histories and Futures, The University of Melbourne, Parkville, Victoria 3010, Australia; i Australian Nuclear Science and Technology Organisation, Lucas Heights, New South Wales 2234, Australia; j Water Quality Centre, 6515 Trent University, 1600 West Bank Drive, Peterborough, Ontario K9L 0G2, Canada; k Earth and Atmospheric Sciences, 1-26 Earth Sciences Building, University of Alberta, Edmonton, Alberta T6G 2E3, Canada; l John de Laeter Centre, 1649 Curtin University, Bentley, Western Australia 6102, Australia

**Keywords:** Mount Lyell, nonferrous mine, smelting, colonization, flotation, deposition modeling, loading, mercury isotopes

## Abstract

The Mount Lyell copper (Cu) mine in Tasmania, Australia, underwent historical operational changes that influenced mercury (Hg) emissions from ore processing and smelting. This study presents the first record of Hg concentrations (Hg_C_) and accumulation rates (Hg_AR_) using sediment cores from four lakes around Mount Lyell. Hg_C_ and Hg_AR_ increased from the 1890s (onset of smelting), peaked from the 1920s (introduction of the flotation processing method), and declined after 1969 (smelter closure). Mercury isotopic signatures confirmed its anthropogenic source. Modeling of Hg deposition vs distance over the period 1922–1969 showed that it followed a power-law function. The Mount Lyell emissions may have affected an area up to ∼270,000 km^2^, beyond which deposition was indistinguishable from the natural background. Estimated total Hg loadings ranged from 23 to 43 t, compared to an estimated ∼150 t Hg contained in the ore floated. Isotopic data showed Δ^199^Hg trending toward zero near the smelter, indicating that the smelter was the main source of Hg. Our findings highlight that pyrometallurgical smelting methods contributed more significantly to Hg emissions than production volume. Studying legacy mines in the Southern Hemisphere, responsible for 29.1% of global Cu production during the preregulatory era (1880–1950), is critical to understanding historical Hg dispersion in this understudied region.

## Graphical abstract

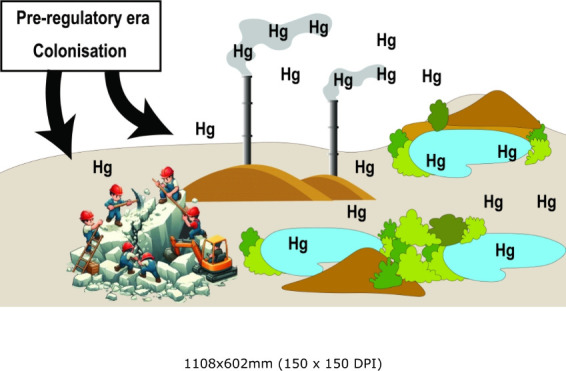

## Introduction

Many copper (Cu) smelters worldwide were established in colonial territories during the 19th and early 20th centuries. The extraction and processing of Cu were integral to those economies.^1^ Colonial powers sought to exploit Cu-rich regions to meet the increasing demand for the metal, which was essential for industrialization and infrastructure development in Europe and North America.^2^

The colonial settlement of Australia in the 1800s resulted in significant environmental changes and metal contamination.^3,4^ This period, coinciding with European industrialization and the exploitation of Australia’s rich minerals, led to substantial increases in energy consumption and carbon emissions.^5,6^ Colonial smelting operations used technologies of the era, many of which were inefficient by today’s standards and led to the unintentional release of pollutants such as mercury (Hg).^7,8^ This issue is further exacerbated by the fact that these operations were established long before the implementation of environmental legislation and pollution control measures.^9^

Due to Mount Lyell’s substantial mineral reserves and the distinctive properties of its ores (i.e., its high reactive pyrite content),^10^ Mount Lyell became one of the most economically viable mining operations globally, sustaining continuous production from 1893 to 1994 ^9^ (noting it was reopened in December 1995 and continued operating until March 2014). Over the 20th century, the site yielded approximately 45 tonnes (t) of gold (Au), 1.3 million t of Cu, and 750 t of silver (Ag).^11^ This success positioned the Mount Lyell Mining and Railway Company (MLMRC) as a leading profitable Cu producer in the British Commonwealth and the largest producer in the Southern Hemisphere in the early 20th century.^12^

While the mining activities in Mount Lyell drove an economic boom and prosperity for Tasmania and its shareholders and investors beyond, they were detrimental to the surrounding environment. The smelting process discharged large amounts of sulfur dioxide which, combined with logging operations, bushfires, and the wet climate, resulted in extensive damage to the local environment, including acid rain and destruction of the local vegetation leaving the hills almost completely bare for many decades to follow.^13–15^

The range of ore processing methods and the scale of mining operations at Mount Lyell changed multiple times throughout its operational history, providing a valuable opportunity to examine Hg emissions associated with different nonferrous mining, processing, and smelting techniques. This distinction highlights the need for research specifically tailored to colonies’ historical and environmental context to comprehensively understand the extent and impacts of Hg pollution from nonferrous metal production associated with colonization.

Legacy Hg emissions from colonial-era activities remain a concern not only at the regional scale but also globally. While oxidized forms of Hg from smelting are quickly removed from the atmosphere through wet and dry deposition, elemental mercury (Hg^0^) can persist much longer, enabling long-range transport and deposition far from the original source.^16^ Lake sediments serve as valuable archives of atmospheric Hg, recording historical trends in deposition. Recent advances in Hg stable isotope analysis have enhanced our ability to trace the source and pathways. The combined analysis of mass-dependent fractionation (MDF) and mass-independent fractionation (MIF) signatures enables differentiation between broad source types such as natural background versus anthropogenic origins. MIF of odd Hg isotopes (^199^Hg and ^201^Hg) can distinguish Hg influenced by atmospheric photochemical processes from that released via direct emissions.^8,17^ When integrated with sediment chronologies, these isotopic tools provide powerful insights into both local and global contributions to Hg contamination.

In this study, we present the first record of Hg concentrations (Hg_C_) and accumulation rates (Hg_AR_) in Tasmania, Australia, derived from four isolated lake sites within small watersheds surrounding Mount Lyell. Lake sediment cores were used to reconstruct historical atmospheric Hg deposition, providing a timeline of natural background variation as well as contamination linked to mining and smelting activities. We analyze temporal Hg_C_ and Hg_AR_ in the context of mining production figures, highlighting the effects of different mining and processing techniques on Hg emission and deposition. We modeled Hg deposition vs distance and obtained a cumulative mass of Hg loading to the environment from the smelting operations. In addition, we conducted isotopic analyses to infer the sources of Hg and the contribution from smelting emission/deposition in the area. Understanding the legacy of Hg pollution from colonization activities in Tasmania and its environmental impact is crucial for assessing Hg cycling and supporting restoration efforts, particularly in the UNESCO Tasmanian Wilderness World Heritage Area.^18^

## Materials and Methods

### Historical Setting

The Mount Lyell Cu–Au–Ag deposit is located within the late Cambrian Mount Read Volcanic deposits in Queenstown, Tasmania. The deposit is notably characterized by its stringer pyrite-chalcopyrite mineralization within quartz-sericite-chlorite rocks.^19^ Detailed information about the local geology and history of mining is available in the Supporting Information.

The smelter, situated approximately 2 km from the mine, began pyritic smelting in July 1896 (Figure 1). In 1916 pyritic smelting transitioned to semipyritic smelting. With the increasing reserves of lower-grade siliceous ore, in 1922, the mine introduced a selective flotation method, which concentrated Cu and enabled the removal of pyrite from the concentrate, significantly improving smelting costs and efficiency.^20^ In December 1969 the smelter closed down, and Cu concentrates were shipped to mainland Australian and overseas smelters^11^ (Figure 1). The mine operated continuously until December 1994, at which time the MLMRC was liquidated and the Tasmanian state accepted environmental liability for the site. The Mount Lyell area has become infamous for its environmental pollution, primarily due to acidic mine drainage, barren landscapes, and the disposal of mine tailings into the King and Queen Rivers.

**Figure 1:**
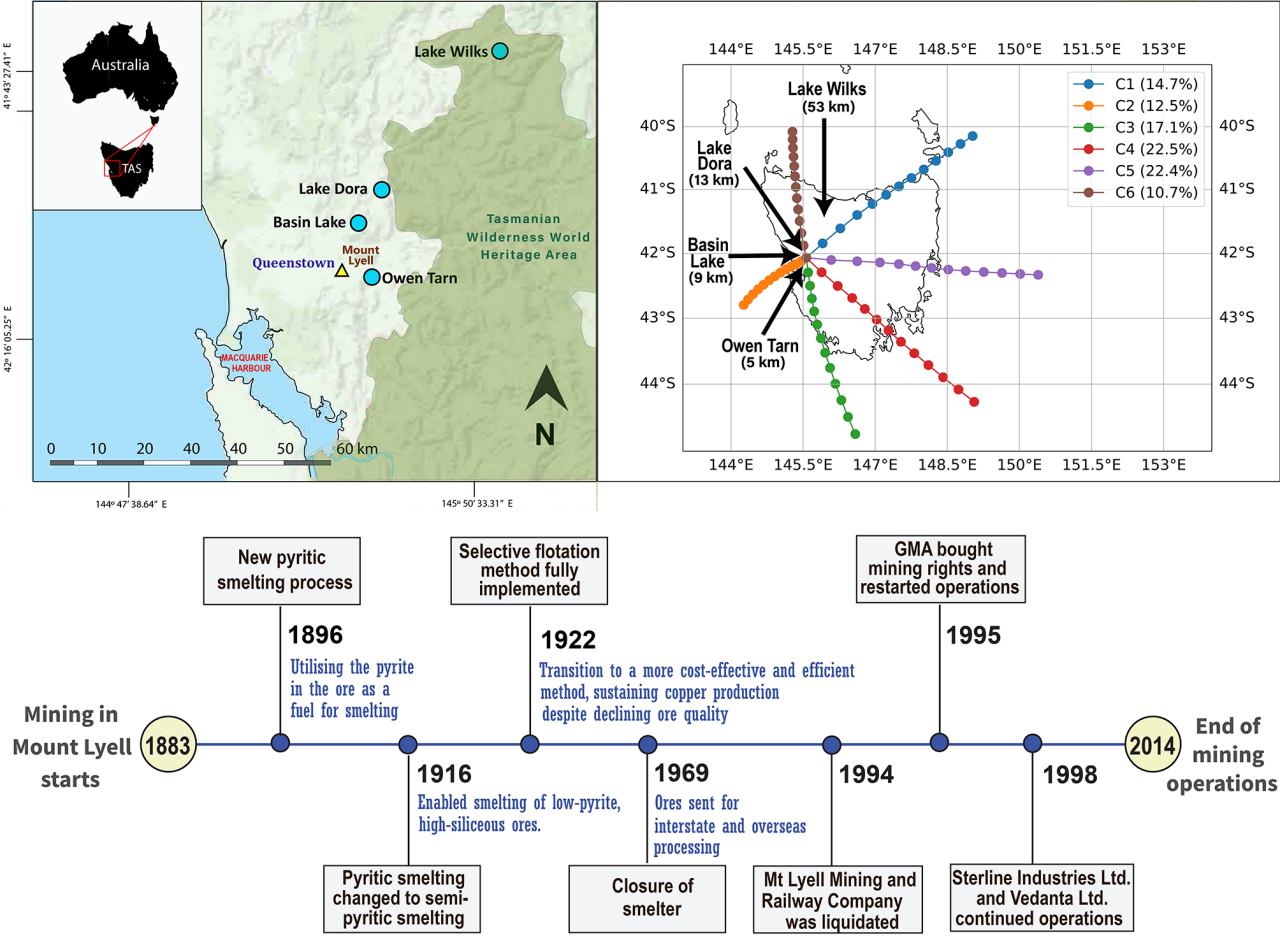
Map of Mount Lyell and surrounding lakes studied in western Tasmania. Arrows label the lakes’ names. Distances between lakes and Mount Lyell are shown in brackets. Left panel: The Tasmanian Wilderness World Heritage Area is highlighted in dark green. The inset map at the top left shows the location of western Tasmania within Tasmania, Australia. Right panel: HYSPLIT model^21,22^ output showing climatological forward wind trajectories from the smelting site, grouped into six clusters to represent the dominant modes of air-mass transport. The average trajectory in each cluster is shown. Each trajectory is 12 h in duration, with dots marked each hour. Bottom panel: Timeline of key events (refer to main text for details).

The mining rights were bought by a new company, Gold Mines of Australia Ltd. (GMA), which restarted operations in December 1995 through a subsidiary Copper Mines of Tasmania Pty Ltd. (CMT; which was exempted from historic environmental liabilities by the Tasmanian Government). After the bankruptcy of GMA in late 1998, CMT was purchased by the Indian company Sterlite Industries Ltd., later to become Vedanta Ltd., and continued operations until mine accidents forced the closure again in March 2014. The site has remained in care and maintenance since by Sibanye Stillwater Ltd.

### Sediment Collection

Four lakes were identified as suitable for this analysis: Owen Tarn (5 km south of Mount Lyell), Basin Lake (9 km northwest of Mount Lyell), Lake Dora (13 km north of Mount Lyell), and Lake Wilks (53 km north of Mount Lyell) (Figure 1). Surface sediment cores (one per lake) were collected from the deepest point of each lake to facilitate these investigations. Details on the site and coring methods are provided in Supporting Information Table 1. After collection, sediment cores were sectioned into 1 cm slices and stored in plastic bags at 4 °C in the Palaeoworks Laboratory at the Australian National University, Canberra.

For calculating Hg background, Hg_C_ and Hg_AR_ were averaged over the period from approximately 1500 to 1800 CE. For historical interpretation, metal concentrations from 1800 to 2014 were considered, and therefore radiometric analysis was used to date sediments.

### Age Dating and Age-Depth Model

Lead-210 samples were processed at the Australian Nuclear Science and Technology Organisation (ANSTO), Sydney, using alpha-particle spectrometry and following methods described by Harrison et al.^23^ Details on the method are presented in the Supporting Information, and data are presented in Supplementary Table 4A and Supplementary Figure 1.

Radiocarbon analyses were processed using Accelerator Mass Spectrometry at ANSTO and DirectAMS Radiocarbon Dating Service laboratory, USA (Supporting Information, Table 2 and Supplementary Table 4B).

The age-depth model was constructed using the R-based Plum package,^24^ which employs a Bayesian framework to integrate measurements of both ^210^Pb and ^226^Ra, using an autoregressive gamma process alongside an assumption of constant ^210^Pb flux to derive robust sediment chronologies.

### Mercury Concentration and Isotope Ratio Analyses

Total Hg analyses were conducted using a Milestone Direct Mercury Analyzer (DMA-80 Tri-Cell; Milestone, Bergamo, Italy) through a sequence of thermal decomposition, amalgamation, and atomic absorption spectrometry (Supporting Information Table 5). A subset of samples (based on remaining sample quantity) from Owen Tarn (n = 9), Basin Lake (n = 2), Lake Dora (n = 6), and Lake Wilks (n = 7) was selected for Hg isotope measurements by a multicollector inductively coupled plasma mass spectrometer (MC-ICP-MS, Neptune) at Trent University, Peterborough, Ontario, according to our previous methods.^8^ More details on the methods are given in the Supporting Information.

### Mercury Contamination Index (Hg_CI_)

The Hg contamination index (Hg_CI_) was calculated by normalizing Hg_C_ to organic matter for each layer and dividing this value by the pre-1800 background Hg-to-organic matter average value. Natural background Hg-to-organic matter values for all lakes were calculated considering the period between 1500 and 1800 CE. In this study we interpreted the Hg_CI_ as <1 = no contamination or depletion; 1 to 5 = low contamination; 5 to 10 = moderate contamination; >10 = severe contamination.

### Airmass Trajectories

The Hybrid Single-Particle Lagrangian Integrated Trajectory model (HYSPLIT),^21,22^ from the Air Resources Laboratory (ARL), was used to calculate representative airmass trajectories downwind from the smelter. HYSPLIT was configured in forward mode with a trajectory release every 6 h, from 1 Jan 2010 to 1 Jan 2020, from a position which is generally within the daytime atmospheric boundary layer at the smelter location (42°04′02.4″ S, 145°33′56.4″ E, 500 m above ground level). More details are given in the Supporting Information.

### Mercury Deposition Modeling

The deposition of Hg emitted from Mount Lyell during the years when flotation was integrated into the ore processing workflow (1922–1969) was modeled using an isotropic power-law function with deposition shadow (Supplementary Figure 6) as developed by Caritat et al.^25^ The power-law deposition model was established for studying airborne metal transport and deposition around the nickel and Cu smelters on the Kola Peninsula (e.g., Boyd et al.^26^). It has also been applied successfully to metal processing centers elsewhere, e.g. in Canada.^27,28^

### Statistical Analyses

All computations investigating correlations were performed using the software R 4.3.1.^29^ Stratigraphic plots were built using the R package ggplot 2 (https://cran.r-project.org/web/packages/ggplot2/index.html).

Paired *t* tests were conducted for each lake to compare Hg_C_ in sediment samples from the premining and postmining periods. A Generalized Linear Model (GLM) was applied to assess the relationship between log-transformed Hg_C_ (log Hg_C_) and log-transformed organic matter (log OM) to evaluate whether the relationship between Hg and OM have changed between pre- and postmine.^30^

## Results and Discussion

### Age-Depth Model and Chronology

For the purpose of assessing the impact of mining activities on Hg deposition, this study focuses on sedimentary records spanning the last 500 years. Given the relatively slow sedimentation rates of Tasmanian lakes, the period of interest is captured within the top 20 cm of sediment cores from each site.

All four lakes (Figure 1) exhibited similar premining (pre-1800) sedimentation rates, ranging from 0.01 to 0.03 cm yr^–1^ (Figure 2). However, post-1800 sedimentation rates varied across sites, reflecting changes in deposition dynamics: Owen Tarn: 0.19 cm yr^–1^; Basin Lake: 0.062 cm yr^–1^; Lake Dora: 0.063 cm yr^–1^; and Lake Wilks: 0.05 cm yr^–1^. A detailed age-depth model, including considerations of the reservoir effect, is provided in the Supporting Information.

**Figure 2:**
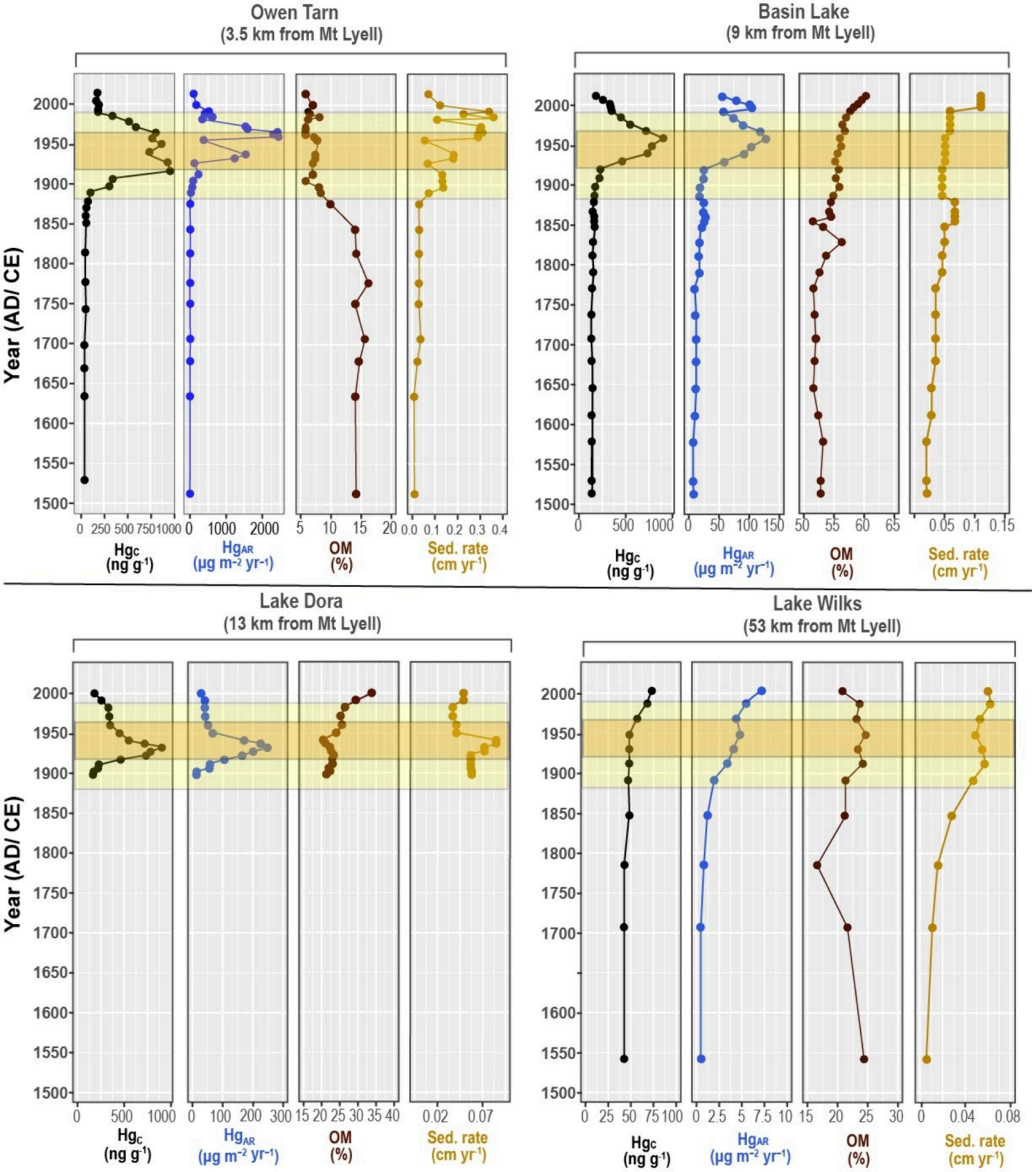
Mercury concentration (Hg_C_), mercury accumulation rate (Hg_AR_), organic matter (OM), and sedimentation rate (Sed. rate) observed in sediment cores from Owen Tarn, Basin Lake, Lake Dora, and Lake Wilks. Note that the *x*-axis scales differ between lakes to optimize visual representation of the data. The period of mining operation at Mount Lyell is colored yellow, while the period of smelter operation using the flotation method is highlighted in orange.

### Mercury Concentration and Accumulation Rates in Lake Sediments Pre- and Postmining

The background profiles of both Hg concentration (Hg_C_) and Hg accumulation rate (Hg_AR_) are unique to each lake, with Basin Lake and Lake Dora presenting a higher background Hg_C_ than Owen Tarn and Lake Wilks (Supplementary Table 3, Figure 2). The background Hg_AR_ (Supplementary Table 3) is consistent with other lake-sediment reconstructions of preanthropogenic Hg deposition in Australia^31,32^ and Southeast Asia.^33^

Paired *t* tests revealed significant differences in Hg_C_ between the pre- and postmining periods for all lakes: Basin Lake, t(13) = 7.8, *p* < 0.001; Owen Tarn, t(8) = 8.5, *p* < 0.001; Lake Dora, t(15) = 6.9, *p* < 0.001; and Lake Wilks, t(4) = 5.9, *p* < 0.05. The Hg_C_ and Hg_AR_ profiles in Owen Tarn, Basin Lake, and Lake Dora are following changes similar to smelting transitions when plotted on their respective age-depth models (Figure 2), with Lake Wilks (53 km north of Mount Lyell) showing a much more subdued Hg_C_ and Hg_AR_ increase compared to the other three lakes (Figure 2). The HYSPLIT results illustrate that while Lake Wilks is not in the main wind direction from Mount Lyell (Figure 1), there is potential for some material to be transported to this lake as it lies closest to the path of the mean trajectory associated with airflow from the south (labeled C6 in Figure 1). This cluster occurs 10.7% of the time and is associated with lower wind speeds, the mean trajectory traveling about 230 km, within 12 h.

In the lakes near the mining site, both Hg_C_ and Hg_AR_ began rising in the late 1800s, coinciding with the onset of mining and smelting activities at Mount Lyell (Figure 2). A pronounced increase in Hg_C_ and Hg_AR_ is observed around 1920, followed by a decline around 1960. This trend aligns with the start of smelting operations in 1896, which peak from the time the flotation method was introduced in 1922 and subsequently decreases after the smelter’s closure in 1969 (Figures 2 and 3).

**Figure 3:**
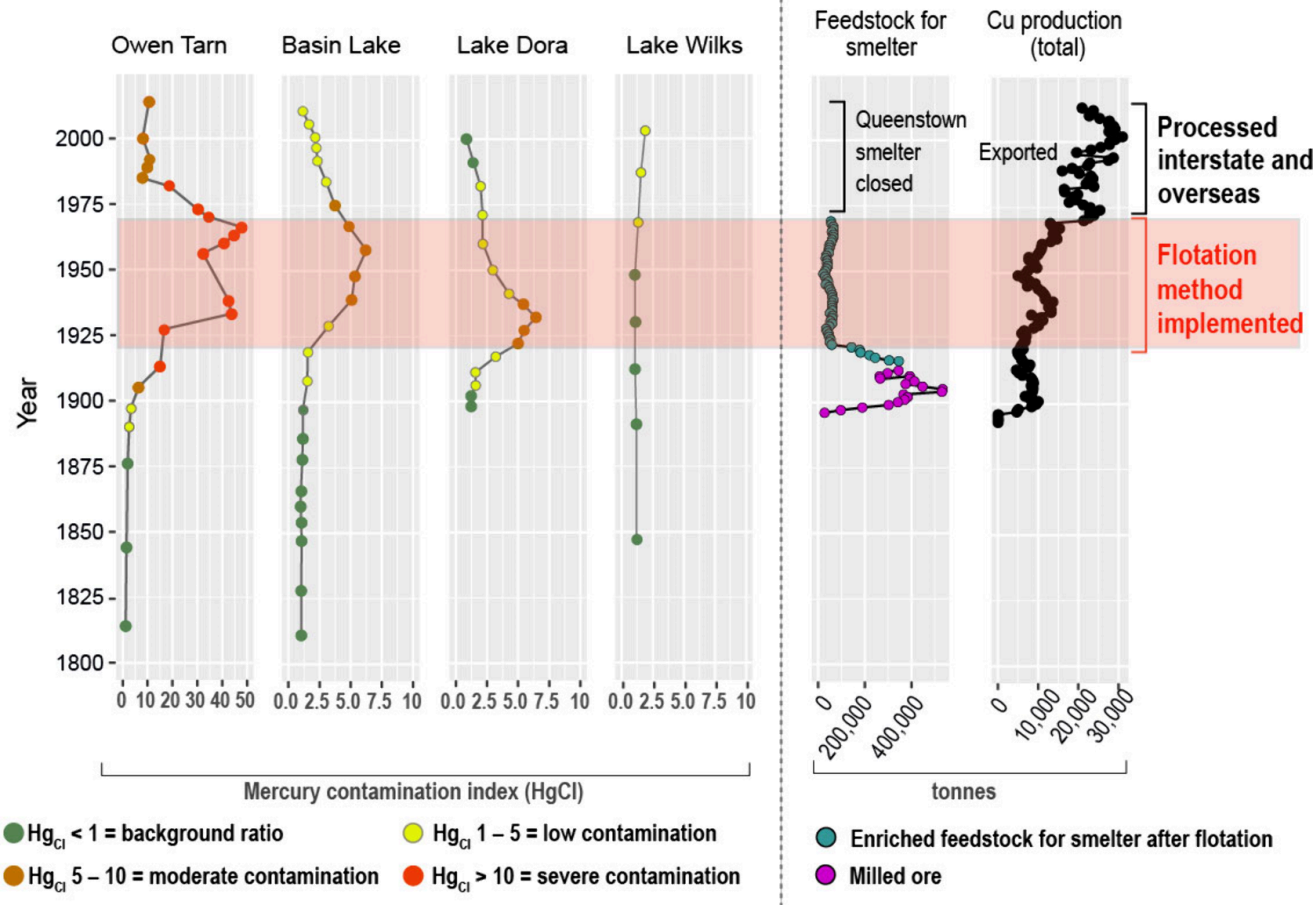
Left: Mercury contamination index (Hg_CI_) in western Tasmanian lake sediments over time. Note the different unit used for Hg_CI_ on the *x*-axis for Owen Tarn, which was adjusted to improve visualization. Right: annual quantity of ore smelted in Mount Lyell and the annual production of Cu (data updated from Mudd, 2007 ^37^).

Notably, neither the amount of ore smelted nor the volume of Cu produced directly correlates with Hg emissions or deposition from the smelter. Instead, the data suggest that pyrometallurgical smelting methods contributed more to Hg emissions than Cu production volume (Figures 2 and 3). This is especially evident during the period in which the flotation method was employed in the smelter. In this process, the Cu-enriched froth from flotation is collected and processed to remove excess moisture, making it more suitable for smelting and refining. The flotation process produces a Cu-rich concentrate low in unwanted gangue and non-Cu-containing sulfide minerals (such as pyrite), and after removal of excess moisture, the concentrate is suitable for smelting.

During flotation, Hg typically follows the Cu-rich sulfide minerals into the concentrate.^34^ Consequently, Hg becomes enriched in the concentrate alongside Cu-bearing sulfide minerals.^34^ This occurs because Hg is often chemically bound to these minerals or exists as a part of hydrophobic, floatable secondary phases. Subsequent to flotation, Hg is volatilized in the Cu smelting furnaces.^35^ In contrast, the fraction of Hg associated with gangue minerals or nonfloatable sulfide phases remains in the tailings.^34^ The distribution of Hg between the concentrate and tailings is influenced by the ore characteristics and the efficiency of the flotation process, although this remains poorly studied globally. Investigating the behavior of Hg in flotation products at Mount Lyell and other Cu mines would provide valuable insights and is an important area for future research.

At Lake Wilks, 53 km north of Mount Lyell, Hg_AR_ increases from ∼1890. This increase in Hg_AR_ is driven mainly by the increase in the sedimentation rate at this time. Hg_C_ began to increase at ∼1950 and continued to rise over time (Figure 2, Supplementary Master Table). The lack of synchrony between Hg_AR_ and Hg_C_ suggests that the observed increase in Hg_AR_ reflects the increased supply of sediment to the lake rather than an increased atmospheric Hg deposition. Lake Wilks exhibits Hg trends consistent with patterns observed elsewhere on mainland Australia, where Hg_C_ begin to rise during the period of the Great Acceleration (∼1950), rather than during the earlier phase of industrialization in the 1800s.^36^

The expansion of West Lyell open-cut mining operations in 1935, combined with the introduction of new machinery and technologies in the 1920s, may have contributed to the release of metals bound to suspended particulate matter in the air. Dust and aerosols generated by these mining processes often carry metals,^38^ potentially influencing the increase in Hg emissions and its subsequent deposition in nearby lakes. However, the data suggest that Hg bound to dust from mining operations was a minor source of Hg to the lakes surrounding Mount Lyell. This conclusion is supported by the significant decline in Hg deposition in the lakes following the smelter’s shutdown in 1969 (Figures 2 and 3), indicating that smelting activities were the dominant source of Hg emissions in the Mount Lyell region. The data specifically suggest that Hg remains associated with the Cu ore post-flotation, leading to higher Hg emissions per unit weight of feedstock postflotation compared to the original ore.

Mount Lyell mining activities had emitted significant amounts of Hg due to the pyrometallurgical smelting, as supported by the elevated Hg_C_ and Hg_AR_ values observed in the lake sediment records (Figures 2 and 3). We acknowledge, however, the inherent complexity of lake–catchment systems, where processes such as erosion, runoff, and dust deposition can introduce secondary variability into sediment records. While these catchment-related influences are recognized and likely present, we consider them insufficient to substantially alter the overall interpretation of Hg emissions reconstructed in this study. The dominant signal preserved in the sediments, particularly in the Hg_CI_ (Figure 3) where Hg is normalized to organic matter, remains consistent with large-scale atmospheric Hg deposition resulting from smelting activities.

### Mercury Deposition Modeling

In order to model Hg deposition using the Caritat et al.^25^ model, the atmospheric dispersion coefficient κ (kappa) for Hg deposition was determined based on the sediment core data from the four lakes around Mount Lyell (Supplementary Master Table). Note that only the Hg accumulation rate above background (i.e., ‘Flotation period (1922–1969) Hg_AR_’ minus ‘Background (before 1800) Hg_AR_’) is modeled to account for the variable nature of processes in the four catchments (see Supplementary Table 3). For those years between 1922 and 1969 that have no measured accumulation rate data (see Supplementary Master Table), Hg_AR_ was linearly interpolated from the bracketing data points. The 1922–1969 above-background Hg deposition-distance power-law regression (Supporting Information Figure 7) yielded a κ value of −2.045 with an r^2^ of 0.96.

Total (distance integrated), cumulative (years 1922 to 1969), above-background (pre-1800 subtracted) deposition, or loading, for Hg was calculated using eq 5 of Caritat et al.^25^ for three values of *x*′, the radius of the shadow zone (0.01, 0.1, and 1 km), and using a *D*_0_ value, the deposition at *x* = 0 (i.e., at the point source), of 1000 kg km^–2^ (where 1 kg km^−2^ = 1000 ug m^−2^). Note that the modeling results are not particularly sensitive to *D*_0_ values, with values half or double the chosen value (thus, 500 and 2000 kg km^–2^) yielding loading results with <10% variance from those reported here once beyond 10 km distance from the source. The total, cumulative, and above-background loading curves as a function of distance, assuming isotropic dispersion, are shown in Figure 4. Semimajor axis length (*L*) of an ellipse with an elongation ratio (*L/l*) of 2 of equivalent area to the modeled circles is also indicated on the figure to show how nonisotropic dispersion may affect downwind transport deposition, increasing transport distance along *L* while reducing it along the ellipse’s semiminor axis (*l*).

**Figure 4:**
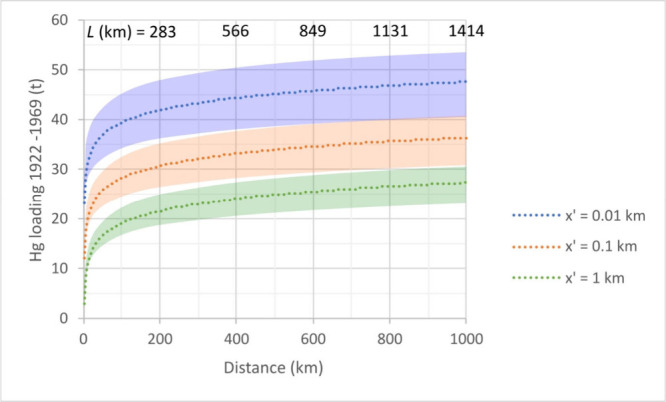
Total (distance integrated), cumulative (years 1922 to 1969), above-background (pre-1800 subtracted) Hg deposition, or Hg loading (t) model results with increasing radius (km) from the Mount Lyell smelter for the isotropic (circular) scenario. For anisotropic scenario, semimajor axis *L* of the equivalent-area ellipse with an elongation ratio (*L/l*) of 2 is shown at the top. Three shadow zone sizes of *x*′ = 0.01, 0.1, and 1 km are shown (blue, upper; orange, middle; and green, lower dotted curves). Uncertainty envelopes around each curve (shaded) represent the standard error (SE) of the predicted loading curve propagated from the variability of the raw data.

The modeling results indicated that

(1) deposition of Hg around Mount Lyell decreased steeply with increasing distance following a power-law function during the flotation period 1922–1969;
(2) mercury deposition reached values indistinguishable from pre-1800 background (∼0.006 kg km^–2^) at radius *R* ∼ 293 km, thus potentially impacting an area up to ∼270,000 km^2^ (equivalent to an ellipse of, for instance, ∼414 km semimajor axis *L* by ∼207 km semiminor axis *l*); and
(3) within that deposition-impacted area, a total mass (loading) of 23 to 43 t (±14% SE) Hg (for *x*′ = 1 and 0.01 km, respectively) may have been added to the background by emissions from the Mount Lyell operations during 1922–1969.

To put these figures of 23–43 t of Hg deposited in perspective, we can compare them with the mass of Hg contained in the processed ore during the same time period. The Mount Lyell mine processed ∼60.2 million t of ore using flotation between 1922 and 1969 with average grades of 0.99% of Cu, ∼0.28 g t^–1^ of Au and ∼1.8 g t^–1^ of Ag to produce 2.13 million t of concentrate containing ∼471,000 t of Cu, 9.66 t of Au, and 58.7 t of Ag (data updated from Mudd, 2007 ^37^). Not much is publicly available in terms of Hg content of Mount Lyell ores, and we had difficulties obtaining ore samples from this specific mine. To overcome this issue, we used the few public-domain ore Hg_C_ values, which are presented in Supporting Information Table 6.

The median Hg_C_ of these Mount Lyell ores is 2.5 mg kg^–1^, and the average concentration is 7 mg kg^–1^. Taking the more conservative (median) value of 2.5 mg kg^–1^, the total amount of Hg present in the ∼60.2 million tons of ore floated at Mount Lyell between 1922 and 1969 would be approximately 150 t. While a portion of this Hg was likely released into the atmosphere as modeled above, other potential Hg sinks at Mount Lyell include waste rock (at least 46 million t by 1969), tailings from flotation-based processing (estimated at 56 million t by 1969), and loss to surface water and groundwater within the local catchment.^39–41^ The exact distribution of Hg among these sinks may not be known with great confidence or at all; however, our findings suggest they are at least of the same order of magnitude as the atmospheric emissions, i.e., at least a few tens of t Hg.

These secondary losses are supported by Hg_C_ reported in Mount Lyell tailings; however, the available data present inconsistencies that require critical evaluation. According to the National Pollutant Inventory (NPI), the average Hg_C_ in Mount Lyell tailings is estimated at approximately 0.05 mg kg^–1^. This value is derived by dividing the total annual Hg reported in tailings by the corresponding mass of ore processed that year. However, this estimate appears implausibly low, equivalent to the average upper continental crustal abundance of Hg (0.05 mg kg^–1^).^42^ This raises questions about the methodology used to generate the NPI data. Notably, this reported value is lower than the pre-1800 background Hg_C_ measured in all lakes included in our study.

In contrast, the Critical Minerals Mapping Initiative (CMMI)^43^ provides a markedly different picture, reporting an average Hg_C_ of 7.5 mg kg^–1^ based on seven tailings ore samples. Within this data set, five samples average 1.3 mg kg^–1^, while two exhibit notably higher concentrations of 17.7 and 28.3 mg kg^–1^. These elevated values suggest a more substantial legacy of Hg retention in mine wastes than indicated by the NPI data and support the likelihood of historical Hg losses during ore processing, especially given the volatilization of Hg during pyrometallurgical smelting.^44,45^

Taken together, these discrepancies highlight the limitations of relying solely on aggregated or self-reported industry data (e.g., NPI) and highlight the need for more independent sample-based characterizations of contaminant concentrations. The higher Hg_C_ values reported by the CMMI provide stronger evidence that significant quantities of Hg remained in the tailings and may have been subsequently mobilized via erosion or leaching, contributing to secondary contamination in surrounding environments.

### Mercury vs Organic Matter

Mining activities have influenced the amount of organic matter (OM) in lakes (Supplementary Figure 8) and its relationship with Hg. For Owen Tarn, the lake closest to the mine, postmining samples showed higher Hg_C_ and lower OM content, with the model exhibiting a strong fit (r^2^ = 0.7, *p* < 0.001) (Supplementary Figure 9). For Basin Lake, prior to mining, OM exhibited no significant correlation with Hg_C_ (r^2^ = 0.05, *p* > 0.05). However, with the onset of mining, a negative relationship was detected for the mining period (r^2^ = 0.37, *p* < 0.05) (Supplementary Figure 9). No significant relationship between OM and Hg_C_ was observed at Lake Dora or Lake Wilks, as indicated by the slope having a p-value > 0.05, both lakes are located further from the mine.

The low OM content in the lakes near the mine can be attributed to the impact of highly sulfur (S_2_)-enriched atmospheric emissions, which increased significantly as the smelting activities intensified. More details are given in the Supporting Information. We note that sedimentation rates in these lakes are low, and the slightly earlier changes in OM relative to the onset of pyritic smelting can be attributed to the low resolution of the sediment core and associated uncertainties in the age–depth model. Nonetheless, the timing and nature of the observed changes in OM are consistent with the onset of pyritic smelting, supporting the interpretation that smelting activities were the primary driver of these changes. This interpretation is further supported by the fact that these lakes are located in remote and previously undisturbed areas of Tasmania, with minimal anthropogenic influence prior to mining activities initiated during colonization.

### Mercury Isotope Ratio Signatures

Analysis of the sediment cores revealed a distinctive isotopic composition, characterized by a progressive increase in Δ^199^Hg (representing MIF) from the background layers to the uppermost sections (Figure 5, Supporting Information Table 8). We only measured the Hg isotope compositions of background sediments in Basin Lake at 21–35 cm (∼847 and 1527 AD/CE), which had comparable Δ^199^Hg (−0.51 ± 0.13‰) as background sediments in Owen Tarn (−0.45 ± 0.06‰) and Lake Dora (−0.53 ± 0.02‰). For Lake Wilks, Δ^199^Hg showed an increase as well. Details are given in the Supporting Information.

**Figure 5:**
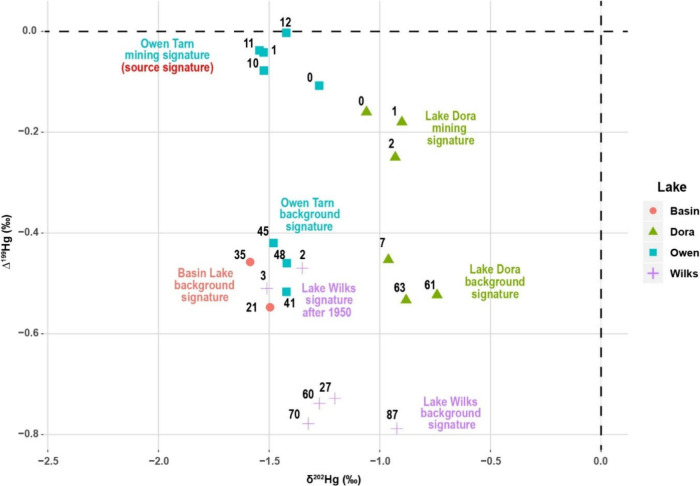
δ^202^Hg vs Δ^199^Hg of lake sediments in four lakes of western Tasmania, Australia (various symbols and colors). Numbers represent the depth of sediment analyzed in the core (cm). See the text for attribution of source signature.

Previous studies showed that the preanthropogenic background sediments are typically characterized by negative Δ^199^Hg,^46,47^ while sediments dominated by anthropogenic Hg contamination have little to no MIF signatures.^48,49^ The observed increases of Δ^199^Hg in the studied lakes, despite differing distances from the smelters, strongly suggest a significant anthropogenic Hg input since the Cu mining operations in Mount Lyell.

In contrast to Δ^199^Hg, δ^202^Hg (representing MDF) did not show distinct trends from the basal layers to the uppermost sections, which we attribute to variable δ^202^Hg in the mined ores, and MDF during processing and refining of ores.^46^ Thus, we use Δ^199^Hg to construct a binary mixing model to quantify the influence of Cu mining operations in Mount Lyell on Hg deposition into lakes

$$\Delta^{199}{\mathrm{Hg}}_{\mathrm{sample}}=f_{\min\mathrm{ing}}\times \Delta^{199}{\mathrm{Hg}}_{\min\mathrm{ing}}+f_{\mathrm{background}}\times \Delta^{199}{\mathrm{Hg}}_{\mathrm{background}}f_{\min\mathrm{ing}}+f_{\mathrm{background}}=1$$

where Δ^199^Hg_sample_, Δ^199^Hg_mining,_ and Δ^199^Hg_background_ denote Δ^199^Hg values of measured sediment samples, Hg deposited from mining operations, and background Hg, respectively. *f*_mining_ and f_background_ are fractional contributions from mining operations and local backgrounds. Here, Δ^199^Hg of the sample from the nearest lake, Owen Tarn, with the highest Hg_C_ (i.e., OT12, Hg_C_ = 935 ng g^–1^, Δ^199^Hg = 0.00 ± 0.07‰, 2SD, Figure 5) is used to represent Δ^199^Hg_mining_ values of Hg deposited from the mining operation. Δ^199^Hg_background_ values are represented by average Δ^199^Hg values of samples from background sediments in individual lakes (Supplementary Table 8). The use of the representative sediment samples rather than the natural and anthropogenic sources as the end-members could greatly eliminate the bias caused by isotope fractionation during Hg depositional and postdepositional processes.

The modeling results show the fractional contributions of mining operations to Hg deposition in lake sediments are 83 ± 5% for Lake Owen Tarn, 58 ± 7% for Lake Dora, and 31 ± 2% (1sd) for Lake Wilks since the 1950s. It is important to note the mining Hg contribution is still as high as ∼80%, even after the cessation of mining and smelting activities, highlighting the legacy Hg left in the surrounding area of Mount Lyell.

### Environmental Implications and Future Directions

The substantial disruption of the natural biogeochemical cycle of Hg and its long-lasting contamination presents a legacy issue dating back to the era of colonial powers, particularly in Australia, Canada, South Africa, and the USA, as well as in regions not directly under colonial rule, such as Wales.^1^ A key question today is how much of the Hg posing environmental concerns globally originates from contemporary activities and how much is a remnant of this historical legacy.

Mercury is a persistent element, and its chemical and physical properties enable it to remain in active circulation once mobilized from geological reservoirs. Once released, it continues to cycle through the atmosphere, ocean, and land for decades to centuries.^50^ As a result, Hg currently depositing into ecosystems from the atmosphere is derived from both modern-day emissions and historical anthropogenic sources, such as smelters operating under colonial powers, which continue to contribute to global cycling. Many of these sites are classified as legacy mines, developed before the establishment of the Environmental Protection Acts, leaving no clear entity accountable for their remediation.

With an estimated affected region of up to ∼270,000 km^2^, equivalent to approximately four times the size of Tasmania, our results suggest that much of Tasmania’s terrestrial landscape, as well as some nearshore marine environments, particularly along the east, north, and south coasts (aligned with dominant wind patterns), may contain evidence of a 20th-century Mount Lyell Hg deposition ‘event’ layer in sediment or more diffuse contamination in soils. Mercury concentration data or Hg isotope analyses in other parts of Tasmania would further validate our findings and provide a clearer picture of the regional extent of Hg emissions.

This study demonstrates the significant contribution of a colonial-era nonferrous mine to atmospheric Hg emissions in the Southern Hemisphere. Mount Lyell is one of several mines established during the preregulatory period to exploit nonferrous metal resources. Our findings indicate that such operations played a key role in shaping the Hg geochemical cycle in the Southern Hemisphere. Between 1880 and 1950, cumulative Cu production (in thousand tonnes, kt Cu) in major Southern Hemisphere countries was as follows: Australia – 1,492.5; Bolivia – 311.8; Chile – 11,024.3; Democratic Republic of Congo – 3,697.6; Namibia – 248.4; Peru – 1,628.1; South Africa – 714.9; and Zambia – 4,113.0.^51^ Together, these countries produced approximately 23,300.9 kt of Cu, representing 29.1% of global production over this period. These figures highlight the role the Southern Hemisphere played in historical nonferrous metal extraction and suggest a substantial, yet largely unquantified, contribution to global Hg emissions from Cu mining and smelting, an area that remains significantly understudied compared to the Northern Hemisphere.

While much of the existing research has focused on colonial mines that directly used Hg (e.g., in Au and Ag amalgamation during Spanish colonization),^52,53^ our study highlights the importance of also examining mines that emitted Hg through other pathways, such as smelting and refining. Expanding the investigation to include additional preregulatory mining, smelting and refining sites across the Southern Hemisphere is essential to fully understanding their contribution to the global legacy Hg pool, which continues to cycle through the environment today.

## Supplementary Material





## Notes

Full data set (Supplementary Master Table) (XLSX)
Detailed information about the local geology and history of mining; details on the method; overview of the freshwater lakes sampled in the study (Supplementary Table 1); radiocarbon results (Supplementary Table 2); Hg accumulation rate above background (Supplementary Table 3); ^210^Pb results (Supplementary Table 4A); radiocarbon (^14^C) age results by sample depth (Supplementary Table 4B); Mercury Standard Reference Material (SRM) and Certified Reference Material (CRM) results and recoveries (Supplementary Table 5); public-domain ore Hg concentration values (Supplementary Table 6); long term Hg isotope compositions and uncertainties for the UM-Almaden standard (Supplementary Table 7); ^199^Hg values of samples from background sediments in individual lakes (Supplementary Table 8); total and supported ^210^Pb activity for lakes studied (Supplementary Figure 1); age-depth model (Supplementary Figures 2–5); power-law deposition model around a point-source emitter (Supplementary Figure 6); 1922-1969 above-background Hg deposition-distance power-law regression (Supplementary Figure 7); percentage of organic matter (OM) in lakes (Supplementary Figure 8); post-mining samples showing higher HgC and lower OM content (Supplementary Figure 9) (PDF)
