# Supplementary material for: The Legacy of Mercury Contamination from Colonial Nonferrous Mining in the Southern Hemisphere
Source: Environ Sci Technol. 2025 Jun 17;59(26):13275–85. doi: 10.1021/acs.est.5c03607 (PMC12243119; doi:10.1021/acs.est.5c03607)
Supplement: Supplementary file 2 [file es5c03607_si_002.pdf]

SUPPLEMENTARY INFORMATION TO:

The Legacy of Mercury Contamination from Colonial Nonferrous Mining in the Southern Hemisphere

Larissa Schneider,* Saul Guerrero, Gavin Mudd, Marco A. A. Lopez, Kristen K. Beck, Ruoyu Sun, Simon G. Haberle, Michael-Shawn Fletcher, Atun Zawadzki, Holger Hintelmann, Alan Griffiths, Colin Cooke, and Patrice de Caritat

https://doi.org/10.1021/acs.est.5c03607

* Larissa.Schneider@anu.edu.au

SUMMARY: 26 PAGES, 9 FIGURES, 8 TABLES.

MATERIALS AND METHODS

Historical Setting

The colonial settlement of Australia in the 1800s resulted in significant environmental changes and metal pollution[1,2]. This period, coincided with European industrialisation and the exploitation of Australia's rich minerals and led to substantial increases in energy consumption and carbon emissions[3]. In many cases, colonial smelting operations used technologies of the era, which were inefficient by today's standards, leading to the unintentional release of pollutants, such as mercury (Hg)[4,5]. This issue is further exacerbated by the fact that these operations were established long before the implementation of environmental legislation and pollution control measures[6].

The Mount Lyell copper (Cu), gold (Au) and silver (Ag) deposit in Tasmania, Australia, gained global recognition for its substantial mineral reserves and the distinctive technical properties of its ores[7]. The Mount Lyell deposit, located at the southern end of the Mount Reid Volcanics in Western Tasmania, hosts disseminated iron (Fe) and Cu sulfide mineralisation. Early mining targeted a massive pyrite deposit known as "The Blow," initially yielding high-grade Au and Ag before transitioning to lower-grade Cu ore[7]. This ore served as a source of iron sulfide (FeS_2), also known as pyrite, for pyritic smelting. The North Lyell mine provided high-grade Cu mineralisation, primarily chalcopyrite ($CuFeS_2$) and bornite (Cu_5FeS_4), which sustained operations through direct smelting and later flotation and roasting processes[7]. The lower-grade disseminated pyrite and chalcopyrite ores were not initially economic but became viable in the 1920s with technological advancements, enabling large-scale exploitation[7].

Although the chalcopyrite and bornite mineralisation initially posed significant challenges for successful smelting, the introduction of the pyritic smelting method transformed smelting in the area into a more efficient and cost-effective process. This approach harnessed the heat generated from the combustion of Fe and S in the pyrite rich ore, significantly reducing the reliance on coke and lowering operational costs[8]. In addition, the initial mining process which relied on direct smelting, later evolved to include gravity concentration and froth flotation[7].

In Mount Lyell, Hg would have emerged as a by-product of Cu smelting. Sulfide ores, such as for Mount Lyell, have high Hg concentrations, which is an effect of Hg's affinity for sulfur and

carbon in certain compounds[5,9]. In the smelting and roasting process to refine Cu of Mount Lyell, Hg would have been emitted as C−Hg and S−Hg bonds are broken and subsequently emitted to the atmosphere as a result of the high vapor pressure of Hg[10]. Given the scale of mining processing in Mount Lyell, a significant amount of Hg is expected to have been emitted during ore processing and smelting operations.

Timeline

The Mount Lyell deposit was discovered in 1883 and was operated by various concerns, firstly as an Au mine but later recognised to be a significant Cu-Au-Ag deposit. The Mount Lyell Company took over the operation in 1893, and active development work and treatment operations began in 1894, continually expanding over time[11].

The smelter was situated at Queenstown one mile from the mines. From 1896 to 1916, 10 blast furnaces treated 1,000 tonnes (t) of ore daily, of an average 2.81 % of Cu, 0.07 kg t^{-1} of Ag and 0.03 kg t^{-1} of Au for the annual production of 8,650 t of blister Cu (averaging 98.8% Cu), 650 kg of Au and 21.7 t of Ag[12]. With the decline in Cu content of the Mount Lyell pyrite to 0.6%, and the emergence of large quantities of siliceous ore, a second phase of smelting commenced. In 1916 pyritic smelting transitioned to semi-pyritic smelting and during this period, three furnaces treated 1,500 t of ore daily, for an annual production of 6,090 t of blister Cu (99.0% Cu), 137 kg of Au and 6.2 t of Ag (data updated from Mudd, 2007[12]).

With the increasing reserves of lower-grade siliceous ore, wet concentration became an essential step in preparing the ore for blast furnace treatment[8]. From 1916, the smelter introduced a selective flotation method, which produced a Cu concentrate and enabled the removal of pyrite from the material to be smelted, significantly improving smelter costs and efficiency[13]. This innovation reduced both flux and fuel consumption while enhancing furnace capacity. After expansion of the flotation circuits to full scale by 1922, a single smelting furnace remained in operation, producing between 7,500 and 15,000 t of blister Cu annually (plus ~190 kg of Au and ~1,100 t of Ag, data updated from Mudd, 2007[12]).

In 1969, due to low prices of Cu and decrease of ore concentrates, the smelter closed down and Cu concentrates were shipped to mainland Australian and overseas smelters[14]. A mine expansion in the early 1970s, based on flotation only, allowed annual production to average 20,000 to 30,000 t of Cu in concentrates (plus ~500 kg of Au and ~3,500 t of Ag, data updated from Mudd, 2007[12]), until closure in December 1994. The smelter produced 661,000 t of Cu, 20.1 t of Au and 511 t of Ag over its lifetime, with total production by December 1994 being 1,201, 000 t of Cu, 32.0 t Au and 624.3 t Ag. In addition, all mining activities had produced at least 46 million t of waste rock by 1969 whilst flotation-based processing produced ~56 million t of tailings (residual minerals after extraction of the Cu concentrate). All data updated from Mudd (2007)[12].

Study site

Western Tasmania is a mountainous area predominantly underlain by intensely folded and faulted Cambrian and pre-Cambrian quartzite rocks and conglomerate units, intersected with highly mineralised volcanic belts[15]. The area includes over 4,000 lakes and tarns, mostly of glacial origin, ranging from highly acidic, dystrophic lakes to ultra-oligotrophic clear water lakes[16]. Rainfall reaches a maximum of 3,400 mm/year and annual temperatures range from 3

to 21 °C, with a mean annual temperature of 11 °C [17]. Mount Lyell reaches 917 m above sea level, with a climate largely controlled by the prevailing westerlies that migrate latitudinally through the seasonal cycle, with west to south-westerly airflow dominating in the winter and west to north westerly airflow dominant in the summer.

Sediment collection

Lake sediments serve as valuable archives for reconstructing historical metal deposition. To better reflect atmospheric deposition relative to catchment-derived Hg inputs, lakes with small catchment areas were specifically targeted, minimising significant geochemical remobilisation from drainage basins. The selected sites were strategically chosen to ensure sufficient spatial coverage for characterising the aerial transport of metals from mining activities and documenting spatial variations in metal deposition downwind of the mining sites.

Sediment cores were collected between 2011 and 2015 using a Universal Percussion Corer (diameter: 6.8 cm; Aquatic Research Instruments). Cores were transported to the University of Tasmania and the Australian National University, where they were stored in cold rooms at 3.5 °C. In the laboratories, cores were split longitudinally using a Geotek Core Splitter and subsampled at 0.5 cm or 1 cm intervals using a custom sediment sampling stage. Subsamples were sealed in airtight WhirlPak® bags and stored at 3.5 °C until analysis.

In 2015, all samples were transported to the Australian National University for dry bulk density analyses determined by subsampling 15 mL of sediment from each core layer, drying the subsample, and calculating the ratio of dry mass to volume. The 15 mL sediment samples were freeze-dried for 72 hours using a Christ Alpha 1–2 LDplus lyophiliser (John Morris Scientific, Sydney, Australia), then stored at room temperature in the dark until further analysis.

Sediment dating

Lead-210 samples were processed at the Australian Nuclear Science and Technology Organisation (ANSTO), Sydney, using alpha-particle spectrometry and following methods described by Harrison et al.[18]. Each dried sediment sample (2 g) was spiked with polonium-209 (^{209}Po) and barium-133 (^{133}Ba) tracers. Each sample was then leached with hot nitric and hydrochloric acids to release polonium and radium. Polonium was autoplated onto silver disks after adding the reducing agent hydroxylammonium chloride. Radium and barium were isolated by co-precipitation and collected as colloidal micro-precipitates of barium sulfate on fine membrane filter papers. The activities of ^{210}Po on the silver disks and ^{226}Ra on the membrane filters were determined by alpha-particle spectrometry. Each membrane filter was also counted by gamma-ray spectrometry to measure the ^{133}Ba tracer activity. Chemical yield recoveries of ^{210}Po and ^{226}Ra were calculated using the recoveries of ^{209}Po and ^{133}Ba tracers, respectively. Unsupported ^{210}Pb activity for each sample was calculated from the activity of ^{210}Po (the proxy for total ^{210}Pb) minus the ^{226}Ra activity (the proxy for supported ^{210}Pb).

Thirty bulk sediment samples from Owen Tarn, Basin Lake, and Lake Wilks were radiocarbon dated at ANSTO, Australia and DirectAMS Radiocarbon Dating Service laboratory, USA (Supplementary Table 2). Lake sediments in Tasmania are susceptible to the lake reservoir effect whereby the lake and atmospheric 14C are not in equilibrium causing bulk sediments to contain “old carbon”[19]. Thus, we can infer this parameter using the information available in the ^{210}Pb dated section of the sediment. The methodology described in Schneider et al. (2020)

provides a way to infer this parameter, allowing for a constant correction for the radiocarbon samples:eight for Owen, 11 for Wilks, and two for Basin (Supplementary Table 4).

The age-depth models (Supplementary Figures 2-5) were constructed using the R-based Plum package [20], which employs a Bayesian framework to integrate measurements of both ^{210}Pb and ^{226}Ra, using an autoregressive gamma process alongside an assumption of constant ^{210}Pb flux to derive robust sediment chronologies. Radiocarbon dates were calibrated with the Southern Hemisphere calibration curve SHcal20[21].

For Lake Wilks, we integrated radiocarbon dates (n = 11) with ^{210}Pb data and, as no reservoir effect was suspected, the radiocarbon dates were used without any reservoir effect correction. In contrast to Lake Dora, where the radiocarbon dates were suspected of being affected by a reservoir effect and no additional dates were available, the age-depth model was developed from ^{210}Pb data only. At Owen Tarn, eight radiocarbon dates indicated a suspected reservoir effect, prompting us to use the Python implementation of Plum, which allowed us to explicitly infer and correct for the reservoir effect in the radiocarbon data. A similar approach was applied for the Basin site, where two radiocarbon dates exhibited suspected reservoir effects.

Organic Matter

Organic matter (OM) was analysed by using weight loss on ignition[22]. Sediment samples were freeze-dried and visible organic materials removed before grinding and sieving to < 0.5 mm. Hydrochloric acid (Sigma-Aldrich 37 % w/w) was introduced to five random sediment samples within each lake core to assess potential reactions indicative of carbonates and left to react for 2 h. Given the absence of discernible reaction, subsequent organic analyses were conducted without prior acid pre-treatment.

Samples were sieved to a < 2 mm sieve and approximately 1 g of each sample was weighed out and heated in a muffle furnace (model CEMLL-SD; Labec, Sydney, Australia) at 550 °C for 6 h. Once cooled to room temperature, samples were weighed, and the difference was reported as organic content in mass/mass percentages (%).

Mercury Concentration and Isotope Ratio Analyses

Sediment samples were stored in clean glass vials, covered with parafilm, placed in a FreeZone Plus 6 freeze-drier (Labconco, Kansas City, MO), and lyophilized at −50 °C for 48 h. Dried samples were then manually homogenised.

Total Hg analyses were conducted using a Milestone Direct Mercury Analyzer (DMA-80 Tri-Cell; Milestone, Bergamo, Italy) through a sequence of thermal decomposition, amalgamation, and atomic absorption spectrometry. The instrument was calibrated with aqueous Hg standard for AAS Sigma Aldrich TraceCERT® (1000 mg/L Hg in nitric acid). A calibration curve was constructed by plotting the absorbances of standards against Hg concentrations in nanograms, which was considered valid for an r^2 value of 0.99 or higher.

Samples were analysed using the USEPA method 7473 (USEPA, 1998); two blanks and two Standard Reference Materials (SRMs) were analysed for every 40 samples. Approximately 100 mg of sample was weighed in nickel boats. After every 10th sample, a replicate sample was

analysed. When replicate recovery variance exceeded 10% compared to the original sample, a third replicate was run. Certified sediment reference materials NIST 2706 (New Jersey soil), NIST 1646a (estuarine sediment), BCR 277R (estuarine sediment), WQB 1 (Lake Ontario sediment) were analysed, and results were in agreement with published values (Supplementary Table 5).

A subset of samples from Owen Tarn (n = 9), Basin Lake (n = 2), Lake Dora (n = 6), and Lake Wilks (n = 7) were selected for Hg isotope measurements at Trent University, Peterborough, Ontario, based on the remaining sample quantities. Approximately 200−600 mg of each selected sample was digested using 10 mL of aqua regia (HNO_3/HCl 1/3 v/v, TraceMetal grade) in a 40 mL glass vessel heated on a hot plate in a clean fume hood [23,24]. The sample was digested at 110−120 °C for a period of ~24 h until ~1 mL of solution remained. During digestion, an acid-cleaned glass marble was fitted to the open glass vessel to create a refluxing condition while allowing acid fumes to be slowly purged out. After digestion, all samples were centrifuged, and the supernatants were brought back to 25 mL volumes with a matrix solution composed of 0.5% BrCl (0.2 M), 0.5% HCl and 0.05% $NH_2OH \cdot HCl$ (20%). The digested solutions were analysed for total Hg concentrations by cold vapor atomic fluorescence spectrometry (CV-AFS, Tekran 2600, US-EPA method 1631E) before the Hg isotope ratio measurement to evaluate the digestion recoveries of Hg, which were in the range of 90−110 % for both the samples and the NIST 1944 standard.

Mercury isotope ratios in the digested solutions (adjusted to Hg concentrations of 1-2 ng g^{-1}) were measured by multicollector inductively coupled plasma mass spectrometry (MC-ICP-MS, Neptune) after matching (±10%) the bracketing NIST 3133 standard solution in both Hg concentration and solution matrix.

The Hg isotope ratio is denoted as $\delta^{xxx}Hg$ (‰, xxx = 199, 200, 201, 202 and 204) by normalizing to the NIST 3133:

$$\delta^{xxx}Hg(‰) = \left(\frac{\left({}^{xxx}Hg/{}^{198}Hg\right)_{sample}}{\left({}^{xxx}Hg/{}^{198}Hg\right)_{NIST\ SRM-3133}} - 1\right) \times 1000 \qquad (1)$$

We use $\delta^{202}Hg$ to denote mass-dependent fractionation (MDF). Mass-independent fractionation (MIF) value is denoted as $\Delta^{xxx}Hg$ (‰, xxx = 199, 200, 201 and 204), representing the difference between the measured $\delta^{xxx}Hg$ value and that predicted from $\delta^{202}Hg$ using a kinetic MDF law [24]:

$$\Delta^{xxx}Hg(‰) = \delta^{xxx}Hg - {}^{xxx}\beta \times \delta^{202}Hg \qquad (2)$$

The mass-dependent scaling factor ${}^{xxx}\beta$ is 0.2520 for ^{199}Hg, 0.5024 for ^{200}Hg, 0.7520 for ^{201}Hg, and 1.4930 for ^{204}Hg.

The typical 2σ analytic uncertainties of our samples were estimated as the 2SD of periodically measured standard UM-Almaden, which yielded mean (±2SD) Hg isotope ratios of -0.58 ± 0.19‰ for $\delta^{202}Hg$, for -0.01 ± 0.04‰ for $\Delta^{199}Hg$, 0.00 ± 0.06‰ for $\Delta^{200}Hg$, and -0.03 ± 0.07‰ for $\Delta^{201}Hg$ (Supplementary Table 7). For samples with replicate analysis, their 2SD values were used as the analytic uncertainties if they are larger than the typical 2σ analytic uncertainties.

Airmass Trajectories

The Hybrid Single-Particle Lagrangian Integrated Trajectory model (HYSPLIT)[25,26], from the Air Resources Laboratory (ARL), was used to calculate representative airmass trajectories downwind from the smelter. HYSPLIT was configured in forward mode with a trajectory release every 6 h, from 1 Jan 2010 to 1 Jan 2020, from a position which is generally within the daytime atmospheric boundary layer at the smelter location (42°04'02.4"S 145°33'56.4"E, 500 m above ground level). Individual trajectories were integrated forward for 12 h using the NCEP/NCAR reanalysis fields, using the files supplied by ARL, with 2.5 ° horizontal (latitude/longitude) resolution and 6 h temporal resolution.

The decade of individual trajectories were then grouped into 6 clusters using the angle-based distance measure implemented in the R package OpenAir[27] to produce a visualisation of the most common synoptically-forced transport pathways. We assume that the trajectory clusters are representative of the climate over the entire period of interest, neglecting the changes in the westerly wind jet which, in this context, are minor [28].

RESULTS AND DISCUSSION

Age-depth model and chronology

Owen Tarn had an average sedimentation of ~0.01 cm yr^{-1} prior to 1800 and 0.19 cm yr^{-1} post-1800. Pb-210 activity reached background in the Plum age-depth model at 15 cm. A reservoir effect was found in the radiocarbon data and a bimodal distribution was identified in the modelled reservoir effect with peaks at ~750 and ~1250 CE. The reservoir effect was modelled in the Plum age-depth model. There are two shifts in sedimentation in the age-depth model at ~18, 11, 5 and 1 cm depth, shifting from a slower (0.01 cm yr^{-1} below 20 cm) to a faster rate, peaking at 0.36 cm yr^{-1} at the depth of 19 cm.

Basin Lake had an average accumulation rate of ~0.02 cm yr^{-1} pre-1800 and 0.062 cm yr^{-1} post-1800. Lead-210 activity reached background at 10 cm, as shown in the Plum age-depth model. A reservoir effect was identified with a mean of ~750 yrs. The reservoir effect refers to the alteration of radiocarbon (^{14}C) ages in samples due to the incorporation of carbon from reservoirs with different ^{14}C levels compared to the atmosphere[19]. To overcome this problem, a modelled reservoir effect was applied to the radiocarbon dates in the Plum model. There is a change in sedimentation rate around 18 cm shifting from a slower rate of 0.02 cm yr^{-1} to a faster rate, peaking at 0.11cm yr^{-1}.

Lake Dora has an average accumulation rate of 0.063 cm yr^{-1} over the past 100 years (compared to 0.01 cm yr^{-1} when considering only ^{14}C dates). The age-depth model produced by Plum using only ^{210}Pb data as the ^{14}C data had a reservoir effect that was unable to be validated in our approach. The resulting age-depth model shows a good replication of the ^{210}Pb data and indicates that the core reached background activity at ~7.5 cm. The accumulation rate changes the most at around 3 cm from ~0.08 to 0.03 cm yr^{-1}.

Lake Wilks had a mean accumulation rate of 0.02 cm yr^{-1} pre-1800, and 0.05 cm yr^{-1} post 1800. The ^{210}Pb shows good overlap with the Plum age-depth model, the model reaches background by 8 cm. Two radiocarbon dates do not agree with the resulting age model at 46 cm and 107

cm. There is a change in sedimentation around 9 cm depth from shifting from slower rate of 0.02 cm yr^{-1} to a slightly faster rate, peaking at 0.06 cm yr^{-1}.

Mercury Contamination Index (Hg_{CI}) and Comparison with Australian and New Zealand Sediment Quality Guidelines

The Hg_{CI} in these lakes showed that they have gone through at least moderate contamination, with exception of Lake Wilks (Figure 3). The Hg enrichment peak is concentrated between 1920 and 1970. In Owen Tarn, Hg underwent severe contamination (Figure 3).

In Australia, the Australian and New Zealand Sediment Quality Guidelines[29] outline the criteria for assessing site contamination and determining the requirements for further investigation, or assessment of risk to determine if any further action is required. This document advises further investigation if sediment metal concentrations are above the default guidelines value (DGV) of 150 ng/g and guidelines value- high (GV-high) level of 1000 ng/g Hg.

In Tasmania, Hg_C in freshwater lake sediments are known to be higher than in other parts of the continent[30]. As a result, the DGV threshold is not a suitable benchmark for comparison. This is evident from the background HgC levels in Lake Dora and Basin Lake, which are 139 ± 10 ng g^{-1} and 143 ± 10 ng g^{-1}, respectively, values that are nearly at the DGV threshold of 150 ng g^{-1}. The GV-high threshold (1000 ng g^{-1}) has not been exceeded in any lakes, though for Owen Tarn and Basin Lakes Hg_C reached values such as 962 and 781 ng g^{-1} (Figure 2).

Mercury vs. Organic matter

Mining activities have influenced the amount of organic matter (OM) in lakes (Supplementary Figure 8) and its relationship with Hg. For Owen Tarn, the lake closest to the mine, regression analysis revealed a statistically significant negative relationship between OM and $Hg_{C,}$ (Supplementary Figure 8 9). Change point analyses revealed no changes through time for OM in this lake. Post-mining samples showed higher Hg_C and lower OM concentrations, with the model exhibiting a strong fit ($r^2 = 0.7$, $p < 0.001$) (Figure 4).

For Basin Lake, change point analyses[31] identified shifts in the mean and variance, identifying key transition points within the dataset. Therefore, two Generalized Linear Models (GLMs) were developed, and the change points coincided precisely with the periods immediately before and during mining activities. Prior to mining, organic matter (OM) exhibited no significant correlation with HgC ($r^2 = 0.05$, $p > 0.05$). However, with the onset of mining, a negative relationship was detected for the mining period ($r^2 = 0.37$, $p < 0.05$) (Figure 4). No significant relationship between OM and Hg_C was observed for Lake Dora and Lake Wilks, as indicated by the slope having a p-value > 0.05, both lakes being located further from the mine.

The low organic matter content in the lakes near mine can be attributed to the impact of highly sulfur (S_2)-enriched atmospheric emissions, which increased significantly as smelting activities intensified. It is likely that the introduction of flotation techniques further altered the chemistry of these sulfides, enhancing their reactivity. During atmospheric transport, S_2 oxidized to sulfur dioxide (SO_2), which was subsequently deposited on surrounding catchments as acid rain.

This acid deposition had a detrimental effect on vegetation and organic-rich topsoil horizons, leading to their progressive degradation[32]. As plant cover declined, the lack of root systems

and organic binding agents caused topsoil erosion, preventing natural regeneration. Over time, this process resulted in the loss of organic matter input into the lake. The extent of these impacts varies between closer and more distant catchments, as the balance between sulfur deposition, erosion, and Hg differs based on proximity to the emission source and local environmental conditions.

Mercury isotope signatures

Analysis of the sediment cores revealed a distinctive isotopic composition, characterised by a progressive increase in Δ^{199}Hg (representing MIF) from the background layers to the uppermost section. In detail, Δ^{199}Hg in nearest Owen Tarn varies from -0.48‰ to -0.42‰ in background sediments at depths of ~41-48 cm (between -1364 and 98 AD/CE), and significantly shifted to less negative values near zero (-0.11‰ to -0.02‰) above depths of 12 cm (~1928 AD/CE). Δ^{199}Hg in Lake Dora ranged from -0.53‰ to -0.52‰ in background sediments at depths of 61~63 cm (undated due to reservoir effect) and decreased to -0.45‰ at 7 cm (1902 AD/CE) to between -0.25‰ and -0.16‰ in the top 2 cm (1960 AD/CE). We only measured the Hg isotope compositions of background sediments in Basin Lake at 21-35 cm (~847 and 1527 AD/CE), which had comparable Δ^{199}Hg (-0.56‰ to -0.46‰) as background sediments in Owen Tarn and Lake Dora. For Lake Wilks, its Δ^{199}Hg showed an increase as well, shifting from between -0.78‰ and -0.73‰ in background sediments at depths of 87 to 27 cm (~ -1273 and -2093 AD/CE) to between -0.51‰ and -0.47‰ in the top 3 cm (~1969 AD/CE). The large negative Δ^{199}Hg values of background sediments in lakes were likely caused by strong MIF occurring in catchment soil (depleting odd isotopes in soils) [33] rather than water column processes (enriching odd isotopes in freshwater water and sediments) [34]. The varying contribution of catchment Hg input might explain the significant Δ^{199}Hg difference of background sediments within and among lakes. Previous studies showed that the pre-anthropogenic background sediments are typically characterized by negative Δ^{199}Hg[35,36], while sediments dominated by anthropogenic Hg contamination have little to no MIF signatures[37,38]. The observed increasing trends of Δ^{199}Hg in the studied lakes with different distances from the smelters strongly suggests a significant anthropogenic Hg input since the Cu mining operations in Mount Lyell.

In contrast to Δ^{199}Hg, δ^{202}Hg (representing MDF) did not show distinct trends from the basal layers to the uppermost sections, which we attribute to variable δ^{202}Hg in the mined ores, and MDF during processing and refining of ores[35]. Thus, we use Δ^{199}Hg to construct a binary mixing model to quantify the influence of Cu mining operations in Mount Lyell on Hg deposition into lakes:

$$\Delta^{199}Hg_{sample} = f_{mining}*\Delta^{199}Hg_{mining} + f_{background}*\Delta^{199}Hg_{background}$$

$$f_{mining} + f_{background} = 1$$

Where $\Delta^{199}Hg_{sample}$, $\Delta^{199}Hg_{mining}$, and $\Delta^{199}Hg_{background}$ denote Δ^{199}Hg values of measured sediment samples, Hg deposited from mining operations, and background Hg, respectively. f_{mining} and $f_{background}$ are fractional contributions from mining operations and local backgrounds. Here, Δ^{199}Hg of the sample from the nearest Lake Owen Tarn with the highest Hg concentrations (i.e., OT12, Hg concentration = 935 ng g^{-1}, Δ^{199}Hg = 0.00±0.07‰, 2SD) is used to represent $\Delta^{199}Hg_{mining}$ values of Hg deposited from the mining operation. $\Delta^{199}Hg_{background}$ values are represented by average Δ^{199}Hg values of samples from background sediments in individual lakes, which are -0.45 ± 0.06‰ for Lake Owen Tarn (i.e., OT41, 45, 48), -0.53 ± 0.02‰ for Lake Dora (i.e., D61, 63), -0.51 ± 0.13‰ for Basin Lake (i.e., BL21, 35) and -0.76

± 0.05‰ for Lake Wilks (i.e., Wi 27, 60, 70, 71, 87)). Because the Hg isotope compositions in sediments have already integrated those of sources, superimposed by potential isotope fractionation during Hg depositional and post-depositional processes, the use of the representative sediment samples rather than the natural and anthropogenic sources as the end-members could greatly decease the uncertainties of source quantification[36,39].

Here, we solve the above equations using a Monte Carlo simulation approach (n = 10,000 times) through the pseudorandom number generation function of the MatLab software[40]. TheΔ^{199}Hg uncertainties associated with both the sources and samples are included. The modelling results show the fractional contributions of mining operations to Hg deposition in lake sediments are 83 ± 5% for Lake Owen Tarn, 58±7% for Lake Dora and 31 ± 2 (1sd) % for Lake Wilks since 1950s. It is important to note the mining Hg contribution is still as high as ~80%, even after the cessation of mining and smelting activities, highlighting the legacy mining now still exports substantial amounts of Hg to ecosystems and the need for comprehensive remediation measures of Hg-contaminated sites.

Supplementary Tables

Supplementary Table 1 – Overview of the freshwater lakes sampled in the study, detailing the collection method, year of sampling, and depth data.

**Core**	**Collection method**	**Geographic coordinates**	**Collection year**	**Core length (cm)**
Owen Tarn	Universal Corer	42°05′58″ S, 145°36′33″ E	2014	68
Basin Lake	Universal Corer	41°58'49.06"S, 145°32'54.67" E	2011	85
Lake Dora	Universal Corer	41°56'49.08"S, 145°38'54.12" E	2000	68
Lake Wilks	Mud-water interface corer (< 60 cm) Livingstone (> 60 cm)	41°40'22.77"S, 145°57'18.23" E	2001	99

Supplementary Table 2 – Radiocarbon results for Owen Tarn, Basin Lake, and Lake Wilks. Sample codes D-AMS were analysed at DirectAMS Radiocarbon Dating Service laboratory and OZU were analysed at Australian Nuclear Science and Technology Organisation. Radiocarbon presented in as ^{14}C age with a 1-sigma error and the associated $\delta^{13}C$ (‰ PDB).

Site	Sample code	Top depth (cm)	^{14}C age ± error (1-sigma)	$\delta^{13}C$ (‰)
Owen	OZU876	18	790 ± 40	-25
Owen	D-AMS 015343	24.5	1,402 ± 25	-29
Owen	D-AMS 010821	34	2,300 ± 28	-26
Owen	D-AMS 015344	40	2,904 ± 24	-37
Owen	D-AMS 010822	45.5	3,332 ± 28	-23
Owen	OZU877	50	5,050 ± 60	-27
Owen	D-AMS 010823	65	6,465 ± 65	-27
Owen	D-AMS 015345	67.5	6,537 ± 31	-33
Owen	OZU878	67.5	7,810 ± 60	-25
Basin	D-AMS 087918	22.0	1,750 ± 25	-27
Basin	D-AMS 087919	34.0	2,470 ± 25	-27
Basin	D-AMS 09582	4.0	4,815 ± 40	-30
Basin	D-AMS 087920	72.0	4,900 ± 30	-28
Basin	D-AMS 005258	83.0	6,447 ± 38	-23
Basin	D-AMS 09590	68.0	8,800 ± 60	-34
Basin	D-AMS 005259	37.0	10,120 ± 57	-32
Basin	D-AMS 005260	59.0	10,952 ± 69	-28
Basin	D-AMS 008243	14	14,268 ± 38	-29
Basin	D-AMS 09588	23.0	16,930 ± 80	-28
Wilks	105878	11	660 ± 25	NA
Wilks	101997	23	1,730 ± 25	NA
Wilks	105879	33	2,260 ± 35	NA
Wilks	101998	46	3,590 ± 25	NA
Wilks	101668	87	3,820 ± 30	NA
Wilks	105874	34	1,770 ± 40	NA
Wilks	105875	107	3,570 ± 45	NA
Wilks	105876	151	5,620 ± 75	NA
Wilks	105877	216	8,150 ± 120	NA
Wilks	101669	261	10,055 ± 45	NA

Supplementary Table 3 – Average mercury concentration (Hg_C in ng g^{-1}), Hg accumulation rate (Hg_{AR} in µg m^{-2} yr^{-1}) and organic matter (%) in sediments of lakes in Western Tasmania, Australia. For full dataset, see Supplementary Master Table.

	Owen Tarn			Basin Lake		
	Background (before 1800)	Flotation period (1922-1969)	Increase (fold)	Background (before 1800)	Flotation period (1922-1969)	Increase (fold)
Hg concentration (ng g^{-1})	48 ± 6	776 ± 206	16	138 ± 6	719 ± 161	5.2
Hg accumulation rate (µg m^{-2} yr^{-1})	6.6 ± 3	1492 ± 966	226	10 ± 3	100 ± 27	10
	**Lake Dora**			**Lake Wilks**		
	Background (before 1880)	Flotation period (1922-1969)	Increase (fold)	Background (before 1880)	Flotation period (1922-1969)	Increase (fold)
Hg concentration (ng g^{-1})	143 ± 10	640 ± 197	4.5	43 ± 0.3	51 ± 5	1.2
Hg accumulation rate (µg m^{-2} yr^{-1})	6.5 ± 1.62	161 ± 76	25	0.6 ± 0.2	4.4 ± 0.3	7.3

Supplementary Table 4A – ^{210}Pb results including depth of sample and thickness by site, density (g cm^{-3}), total ^{210}Pb (Bq kg^{-1}) and supported Pb (^{226}Ra Bq kg^{-1}) and their associated standard deviation.

^{210}Pb data							
Site	Depth (cm)	Density (g cm^{-3})	^{210}Pb (Bq kg^{-1})	sd (^{210}Pb)	Thickness (cm)	226Ra (Bq kg^{-1})	sd (^{226}Ra)
Lake Wilks	0.5	0.1	638	28	0.5	43	3
Lake Wilks	1	0.08	463	20	0.5	43	3
Lake Wilks	1.5	0.16	396	18	0.5	43	4
Lake Wilks	2	0.18	361	16	0.5	40	3
Lake Wilks	2.5	0.15	321	14	0.5	40	3
Lake Wilks	3	0.15	257	12	0.5	37	3
Lake Wilks	3.5	0.16	199	9	0.5	35	3
Lake Wilks	4.5	0.18	101	4	0.5	35	3
Lake Wilks	5.5	0.15	76	3	0.5	35	3
Lake Wilks	6.5	0.15	59	3	0.5	35	3

Lake Wilks	7.5	0.15	51	2	0.5	33	3
Lake Wilks	8.5	0.16	56	3	0.5	32	3
Lake Wilks	10	0.14	50	2	0.5	31	2
Lake Dora	0.25	0.45	337	9	0.25	Not available	Not available
Lake Dora	0.5	0.45	313	9	0.25	Not available	Not available
Lake Dora	0.75	0.45	308	12	0.25	Not available	Not available
Lake Dora	1	0.45	294	7	0.25	Not available	Not available
Lake Dora	1.25	0.45	258	6	0.25	Not available	Not available
Lake Dora	1.5	0.45	247	7	0.25	Not available	Not available
Lake Dora	1.75	0.45	194	4	0.25	Not available	Not available
Lake Dora	2	0.45	165	4	0.25	Not available	Not available
Lake Dora	2.25	0.45	138	5	0.25	Not available	Not available
Lake Dora	2.5	0.45	110	3	0.25	Not available	Not available
Lake Dora	2.75	0.45	80	3	0.25	Not available	Not available
Lake Dora	3	0.45	81	3	0.25	Not available	Not available
Lake Dora	3.25	0.45	39	2	0.25	Not available	Not available
Lake Dora	3.5	0.45	30	2	0.25	Not available	Not available
Lake Dora	3.75	0.45	17	2	0.25	Not available	Not available
Lake Dora	4	0.45	18	2	0.25	Not available	Not available
Lake Dora	4.25	0.45	16	2	0.25	Not available	Not available
Lake Dora	4.5	0.45	35	2	0.25	Not available	Not available
Lake Dora	4.75	0.45	30	2	0.25	Not available	Not available
Lake Dora	5	0.45	20	2	0.25	Not available	Not available
Lake Dora	5.25	0.45	16	3	0.25	Not available	Not available
Lake Dora	5.5	0.45	20	3	0.25	Not available	Not available
Lake Dora	5.75	0.45	17	2	0.25	Not available	Not available

Lake Dora	6	0.45	13	3	0.25	Not available	Not available
Lake Dora	6.25	0.45	8	3	0.25	Not available	Not available
Lake Dora	6.5	0.45	10	2	0.25	Not available	Not available
Lake Dora	6.75	0.45	9	2	0.25	Not available	Not available
Lake Dora	7	0.45	11	2	0.25	Not available	Not available
Lake Dora	7.25	0.45	6	2	0.25	Not available	Not available
Lake Dora	7.5	0.46	8	2	0.25	Not available	Not available
Lake Dora	7.75	0.46	7	2	0.25	Not available	Not available
Owen Tarn	0.5	0.63	59	2	0.5	5.7	0.6
Owen Tarn	1.5	1.01	16	0.8	0.5	3.2	0.3
Owen Tarn	4	1.02	8	0.5	0.5	3.7	0.4
Owen Tarn	5.5	0.96	11	0.7	0.5	3.2	0.4
Owen Tarn	12	0.74	9	0.7	0.5	2	0.3
Owen Tarn	14.5	0.6	8	0.7	0.5	3.5	0.5
Owen Tarn	20.5	0.61	8	0.8	0.5	6.6	0.8
Owen Tarn	25.5	0.67	9	0.5	0.5	5.9	0.6
Basin Lake	0.5	0.17	472	21	0.5	5.3	0.6
Basin Lake	2.5	0.21	386	18	0.5	8	2
Basin Lake	4.5	0.25	133	6	0.5	8.4	1.1
Basin Lake	6.5	0.22	53	2	0.5	8	2
Basin Lake	8.5	0.24	13	1	0.5	5.2	0.6
Basin Lake	10.5	0.25	10	1	0.5	5.9	0.7
Basin Lake	14.5	0.25	6	0.5	0.5	4.3	0.5

Supplementary Table 4B. Radiocarbon (^{14}C) age results by sample depth.

^{14}C	Depth (cm)	^{14}C Age	Error
Lake Wilks	11.5	660	25
Lake Wilks	23.5	1730	25
Lake Wilks	33.5	2260	35
Lake Wilks	34.5	1770	40
Lake Wilks	46.5	3590	25
Lake Wilks	87.5	3820	30
Lake Wilks	107.5	3570	45
Lake Wilks	151.5	5620	75
Lake Wilks	216.5	8150	120
Lake Wilks	261.5	10055	45
Owen Tarn	18.5	65	2.5
Owen Tarn	18	790	40
Owen Tarn	24.5	1402	25
Owen Tarn	34	2300	28
Owen Tarn	40	2904	24
Owen Tarn	45.5	3332	28
Owen Tarn	50	5050	60
Owen Tarn	65	6465	31
Owen Tarn	67.5	6537	34
Owen Tarn	67.5	7810	60

Supplementary Table 5 – Mercury Standard Reference Material (SRM) and Certified Reference Material (CRM) results and recoveries. Materials used here are: NIST 1646a (estuarine sediment); NIST 2706 (New Jersey soil); WQB 1 (Lake Ontario sediment); and

BCR-277R (estuarine sediment). SD = Standard Deviation, RSD = Relative Standard Deviation.

SRM/CRM	Results	Reference value	Recovery
	ng g^{-1}		%
BCR-277R	130	128	101
BCR-277R	128		100
BCR-277R	127		99
BCR-277R	127		99
BCR-277R	129		101
BCR-277R	128		100
BCR-277R	127		99
BCR-277R	127		99
**AVERAGE**	**128**		
**SD**	**1.00**		
**RSD**	**0.78**		
WQB-1	1008	1060	95
WQB-1	1031		97
WQB-1	1108		105
WQB-1	1021		96
WQB-1	1080		102
WQB-1	1071		101
WQB-1	1038		98
WQB-1	1069		101
WQB-1	1082		102
WQB-1	1063		100
WQB-1	1062		100
WQB-1	1073		101
**AVERAGE**	**1059**		
**SD**	**$29**		
**RSD**	**2.71**		
NIST 2706A	135	132.9	102
NIST 2706A	134		101
NIST 2706A	134		101
NIST 2706A	133		100
NIST 2706A	134		101
**AVERAGE**	**134**		
**SD**	**0.82**		
**RSD**	**0.61**		
NIST1646a	36	40	90
NIST1646a	37		92
**AVERAGE**	**36**		
**SD**	**0.73**		
**RSD**	**2.01**		

Supplementary Table 6 – Reported Hg concentration in Mount Lyell ore minerals.

Mineral/Ore type	Hg concentration (mg kg^{-1})	Source

Massive hematite–barite	0.7	Denwer (2018)[41], table 2
Massive pyrite	2.5	Denwer (2018)[41], table 2
High-grade Cu–Ag ore	13.2	Denwer (2018)[41], table 2
High-grade Pb–Zn ore	8.6	Denwer (2018)[41], table 2
Massive pyrite	28.3	Champion et al. (2021)[42]
Sphalerite-galena-pyrite	17.7	Champion et al. (2021)[42]
Bornite breccia	3.02	Champion et al. (2021)[42]
Chalcopyrite-pyrite	1.12	Champion et al. (2021)[42]
Chalcopyrite-pyrite	1.0	Champion et al. (2021)[42]
Pyrite-chalcopyrite	0.84	Champion et al. (2021)[42]
Pyrite-chalcopyrite	0.51	Champion et al. (2021)[42]

Supplementary Table 7 - Long term Hg isotope compositions and uncertainties for the UM-Almaden standard. Values are expressed in delta notation (‰), relative to the NIST 3133 Hg standard solution.

Data Analysed	δ^{199}Hg	δ^{200}Hg	δ^{201}Hg	δ^{202}Hg	δ^{204}Hg	Δ^{199}Hg	Δ^{200}Hg	Δ^{201}Hg	Δ^{204}Hg
December 14, 2015	-0.14	-0.30	-0.49	-0.61	-0.82	0.02	0.01	-0.03	0.09
December 22, 2015	-0.13	-0.27	-0.40	-0.56	-0.83	0.02	0.01	0.02	0.01
February 24, 2016	-0.21	-0.36	-0.56	-0.65	-0.86	-0.05	-0.03	-0.07	0.12
February 24, 2016	-0.17	-0.34	-0.54	-0.63	-0.93	-0.02	-0.03	-0.07	0.02
February 24, 2016	-0.22	-0.38	-0.59	-0.71	-0.86	-0.04	-0.02	-0.06	0.20
February 24, 2016	-0.14	-0.29	-0.32	-0.44	-0.73	-0.02	-0.07	0.02	-0.06
February 24, 2016	-0.16	-0.28	-0.46	-0.55	-0.81	-0.02	0.00	-0.04	0.01
March 1, 2016	-0.16	-0.31	-0.55	-0.63	-1.07	0.00	0.01	-0.08	-0.13
March 1, 2016	-0.14	-0.22	-0.39	-0.47	-0.84	-0.02	0.01	-0.04	-0.15
March 1, 2016	-0.13	-0.25	-0.38	-0.51	-0.72	0.00	0.01	0.00	0.04
March 1, 2016	-0.21	-0.36	-0.61	-0.76	-1.20	-0.02	0.02	-0.04	-0.06
March 1, 2016	-0.14	-0.27	-0.56	-0.62	-1.04	0.01	0.05	-0.09	-0.11
March 1, 2016	-0.11	-0.19	-0.34	-0.44	-0.67	0.00	0.03	-0.01	-0.02
March 9, 2016	-0.17	-0.26	-0.44	-0.59	-0.77	-0.02	0.03	0.00	0.10
Average	-0.16	-0.29	-0.47	-0.58	-0.87	-0.01	0.00	-0.03	0.00
SD	0.03	0.06	0.10	0.10	0.15	0.02	0.03	0.04	0.10

Supplementary Table 8 – Overview of mercury (Hg) isotope data and associated parameters across sediment cores.

Sample	Date Analysed	AD/CE	Hg (ng/g)	Hg flux (ug/m2/yr)	Δ^{199}Hg	2sd	Δ^{200}Hg	2sd	Δ^{201}Hg	2sd	Δ^{204}Hg	2sd	
OT0	February 24, 2016	2015	183.4	102.2	-0.11		0.08		-0.10		-0.07		
OT1	February 24, 2016	2001	170.1	176.6	-0.02		0.06		-0.04		-0.11		
OT2	February 24, 2016	1993	199.3	522.4	-0.06		-0.03		-0.01		-0.03		
OT10	February 24, 2016	1960	870.6	2444.4	-0.09	0.02	-0.01	0.11	-0.06	0.01	0.00	0.13	
OT11	February 24, 2016	1957	740.8	378.7	-0.04	0.00	0.01	0.01	0.02	0.13	0.05	0.01	
OT12	February 24, 2016	1939	935.1	1542.4	0.00	0.07	0.00	0.08	0.00	0.01	-0.02	0.19	Mining end-member
OT41	February 24, 2016	-4.43	30.2	1.4	-0.48	0.48	-0.09	0.01	-0.19	0.09	0.02	0.29	background
OT45	February 24, 2016	-443.76	27.7	0.8	-0.42		0.03		-0.07		0.02		background
OT48	February 24, 2016	-1367.07	23.4	0.3	-0.46		0.02		-0.09		-0.08		background
BL21	February 24, 2016	1527.36	135.2	7.0	-0.56	0.01	0.01	0.03	-0.36	0.02	-0.03	0.30	background
BL35	February 24, 2016	847.10	159.3	7.4	-0.46		0.03		-0.27		-0.02		background
D0	February 24, 2016	2000.00	181.4	18.6	-0.16		0.01		-0.08		0.04		
D1	February 24, 2016	1981.47	330.3	40.6	-0.18		-0.03		-0.17		0.04		
D2	March 1, 2016	1956.07	349.6	37.0	-0.25		0.02		-0.24		0.05		
D7	March 1, 2016	1874.46	173.0	31.9	-0.45		0.01		-0.40		-0.12		
D61	March 1, 2016	-1434.13	153.7	7.4	-0.52		0.01		-0.47		-0.06		background
D63	March 1, 2016	-1531.89	161.4	11.1	-0.53		0.00		-0.45		0.01		background
Wi1	March 1, 2016	2004	73.2	7.2	-0.49		0.04		-0.49		0.28		
Wi2	March 1, 2016	1988	68.2	5.5	-0.47		0.01		-0.48		-0.09		
Wi3	March 1, 2016	1969	57.1	4.4	-0.51		0.03		-0.46		-0.18		
Wi27	March 1, 2016	430.80	34.5	1.3	-0.73		-0.01		-0.62		0.09		background
Wi60	March 1, 2016	-984.40	32.3	1.3	-0.74		0.05		-0.64		-0.07		background
Wi70	March 1, 2016	-1395.60	43.9	1.8	-0.78		0.05		-0.65		0.14		background
Wi87	March 1, 2016	-2093.30	44.7	1.5	-0.79		-0.01		-0.68		0.02		background

Note: Highlighted mercury isotope values, representing local background (green) and mining (yellow), were used for source apportionment based on the mercury isotope mixing model.

Supplementary Figures

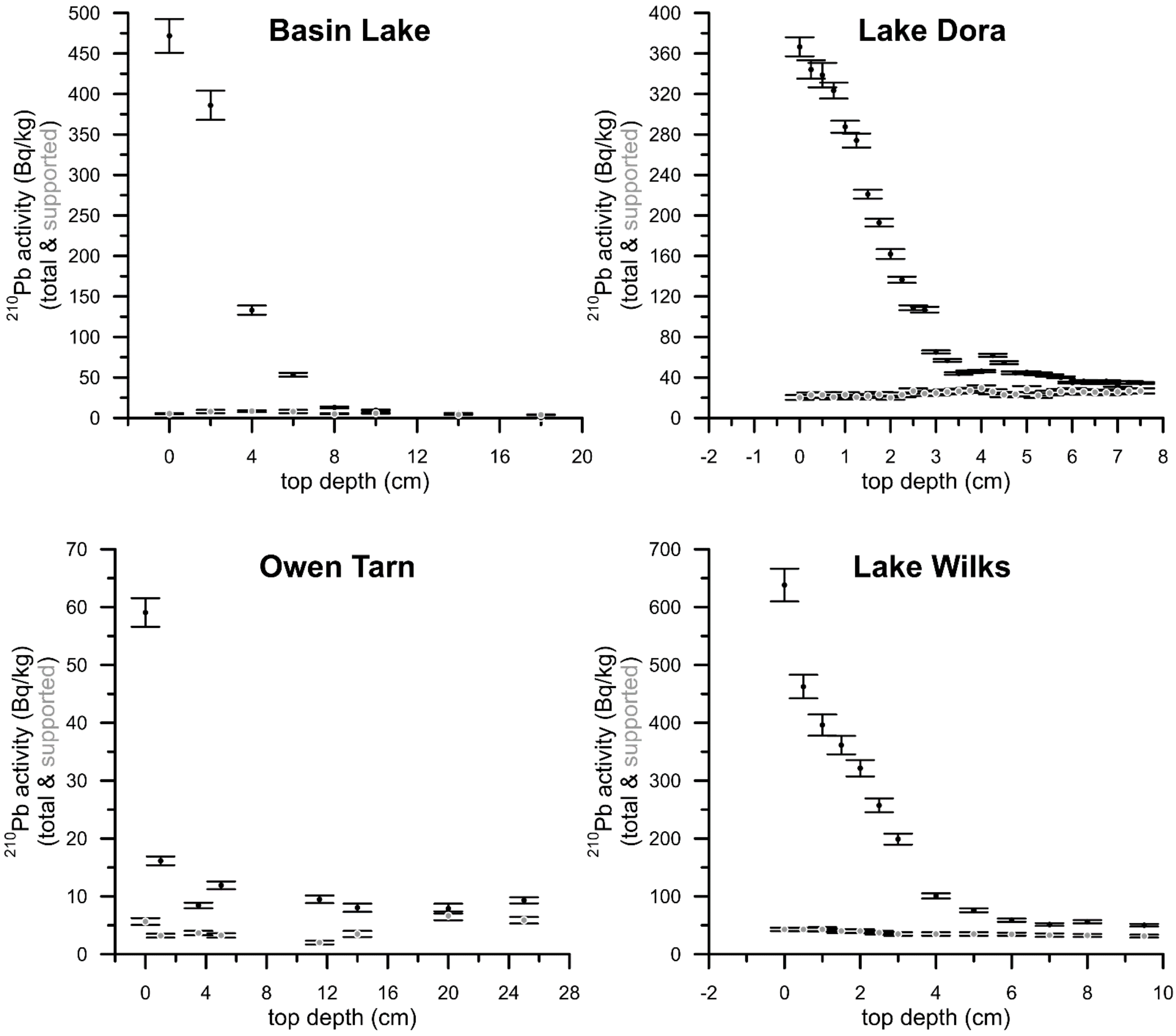

Supplementary Figure 1: Total and supported ^{210}Pb activity (Bq/kg) for the lakes studied.

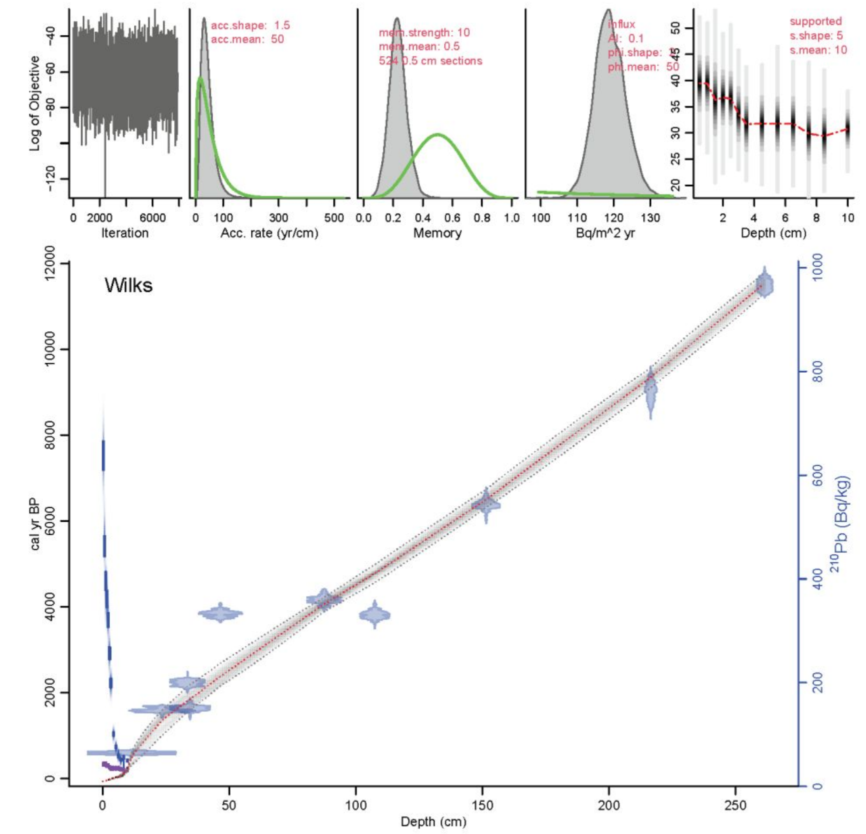

Supplementary Figure 2: Age-depth model for Lake Wilks produced using *plum* in R.

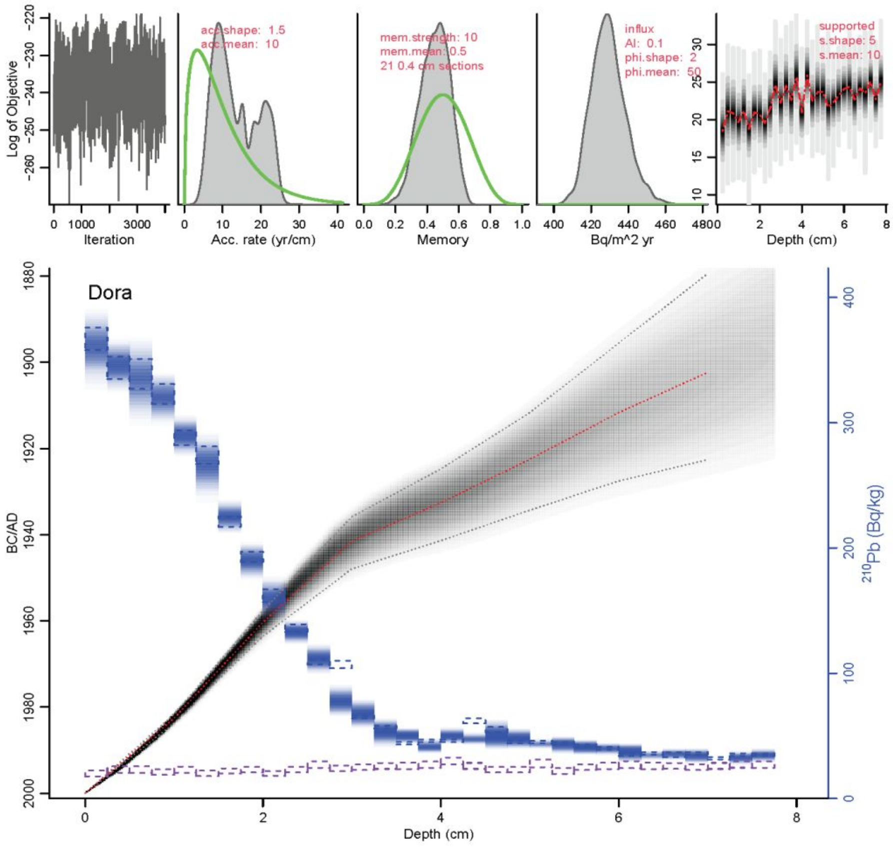

Supplementary Figure 3: Age-depth model for Lake Dora produced using *plum* in R.

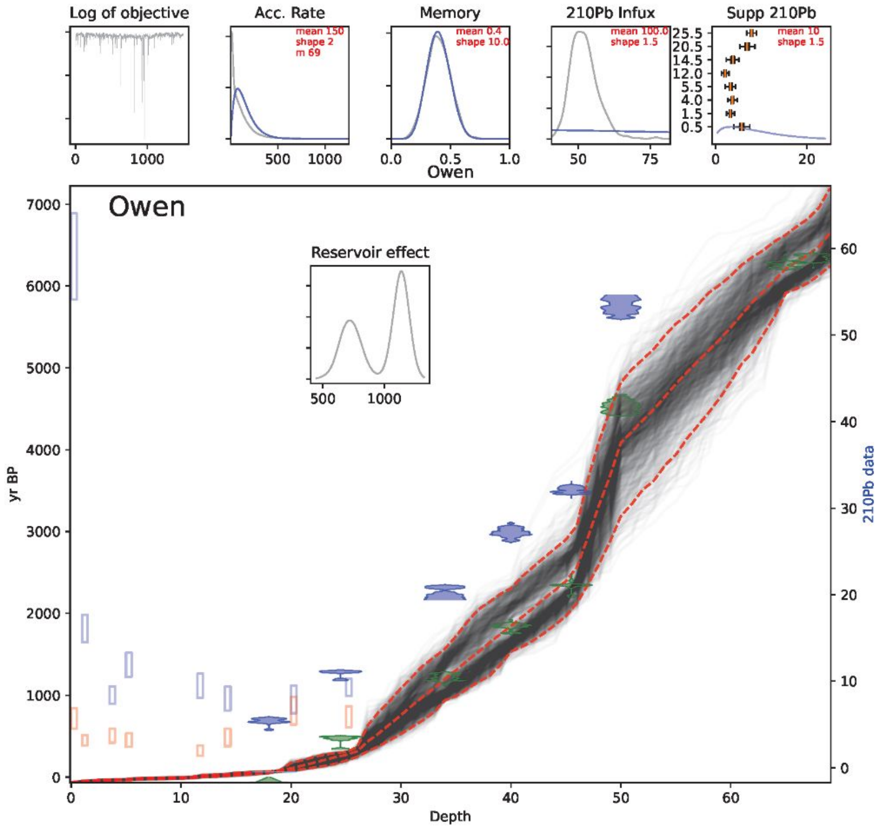

Supplementary Figure 4: Age-depth model for Owen Tarn produced using *plum* in python.

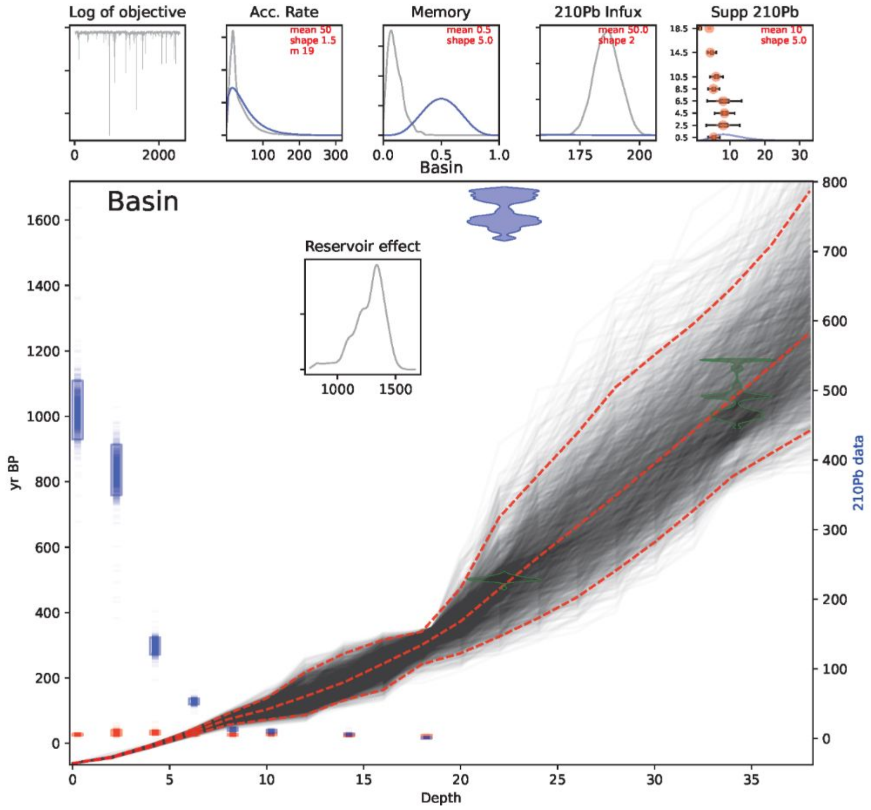

Supplementary Figure 5: Age-depth model for Basin Lake produced using *plum* in python.

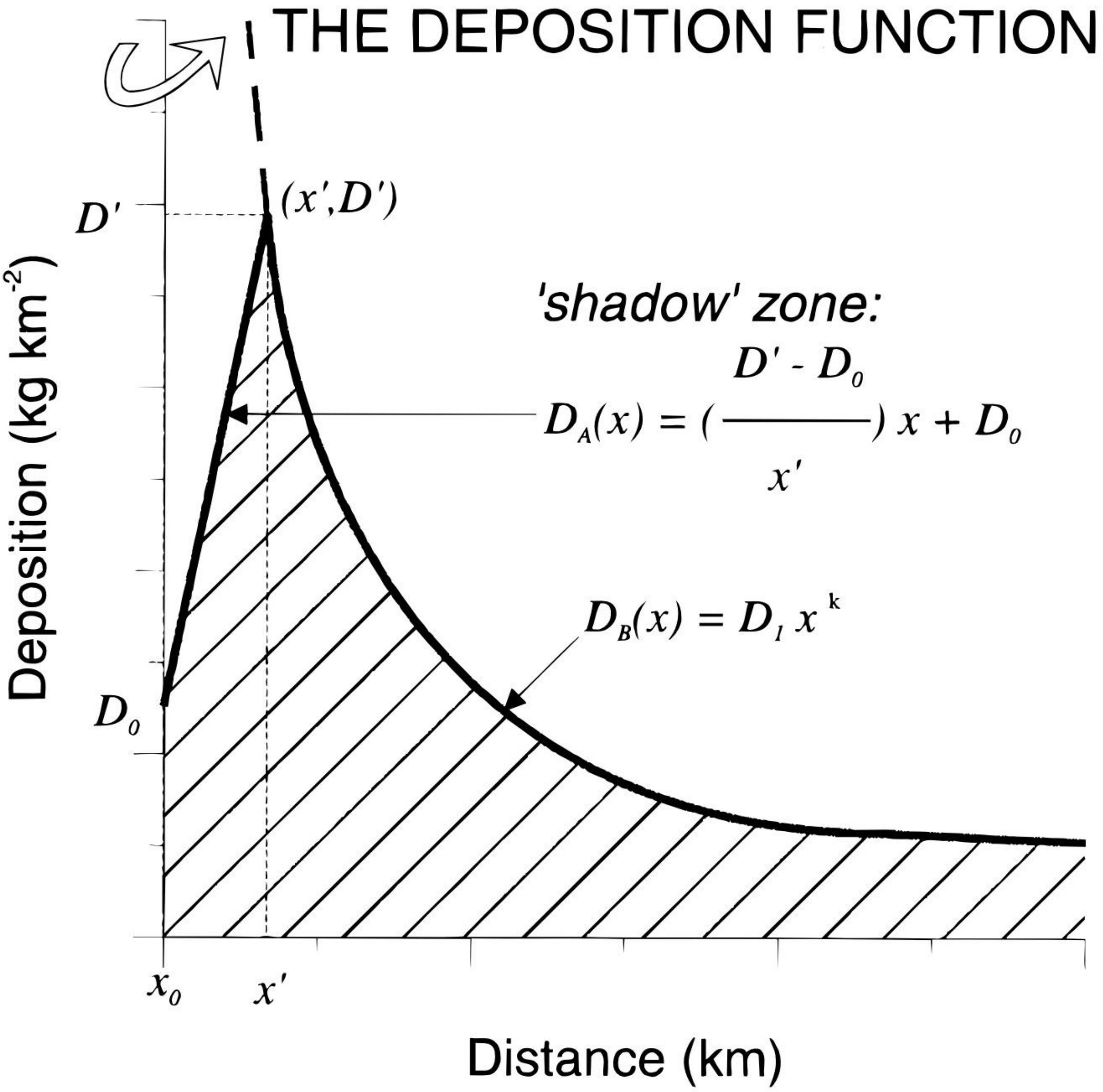

Supplementary Figure 6: Power-law deposition model around a point-source emitter, including a shadow zone of radius *x'* proximal to the smokestack at distance x_0. Reprinted with permission from Caritat et al [43]. Copyright 1997 American Chemical Society.

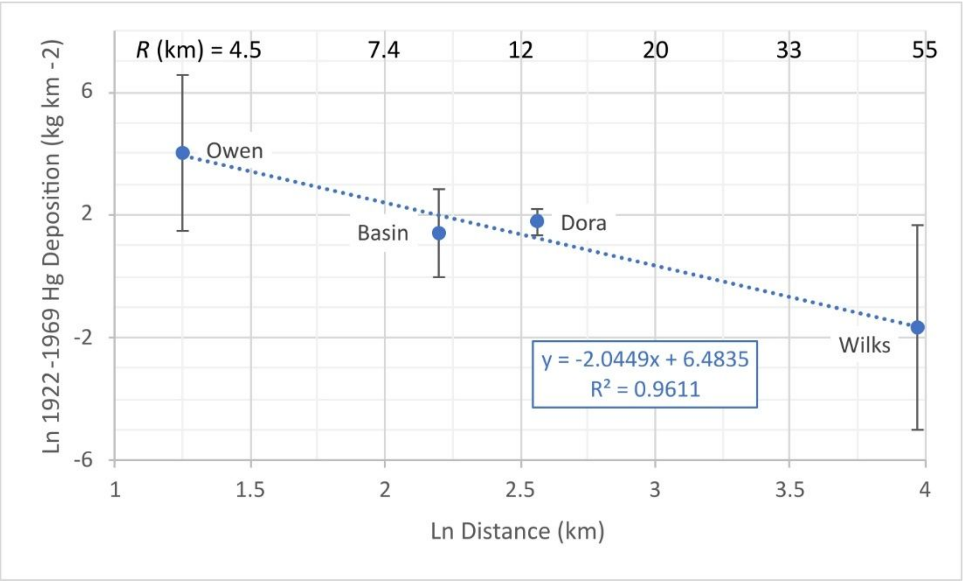

Supplementary Figure 7: Cumulative (1922 to 1969), above-background (pre-1800 deposition subtracted) Hg deposition (kg km^{-2}) at four lakes (Owen Tarn, Basin Lake, Lake Dora, and Lake Wilks) versus distance (km) from Mount Lyell smelter. Power-law regression line and equation are also shown. Error bars represent standard error of the raw data. Note Ln-transformed abscissa and ordinate data. Untransformed radii *R* (km) shown at top.

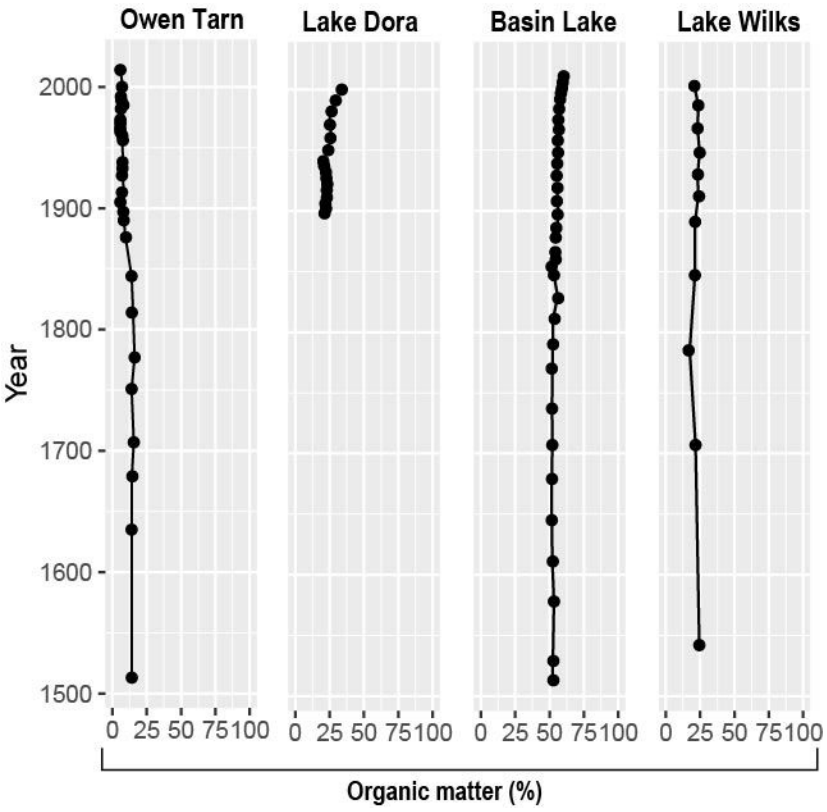

Supplementary Figure 8: Percentage of organic matter in sediments from Owen Tarn, Lake Dora, Basin Lake, and Lake Wilks.

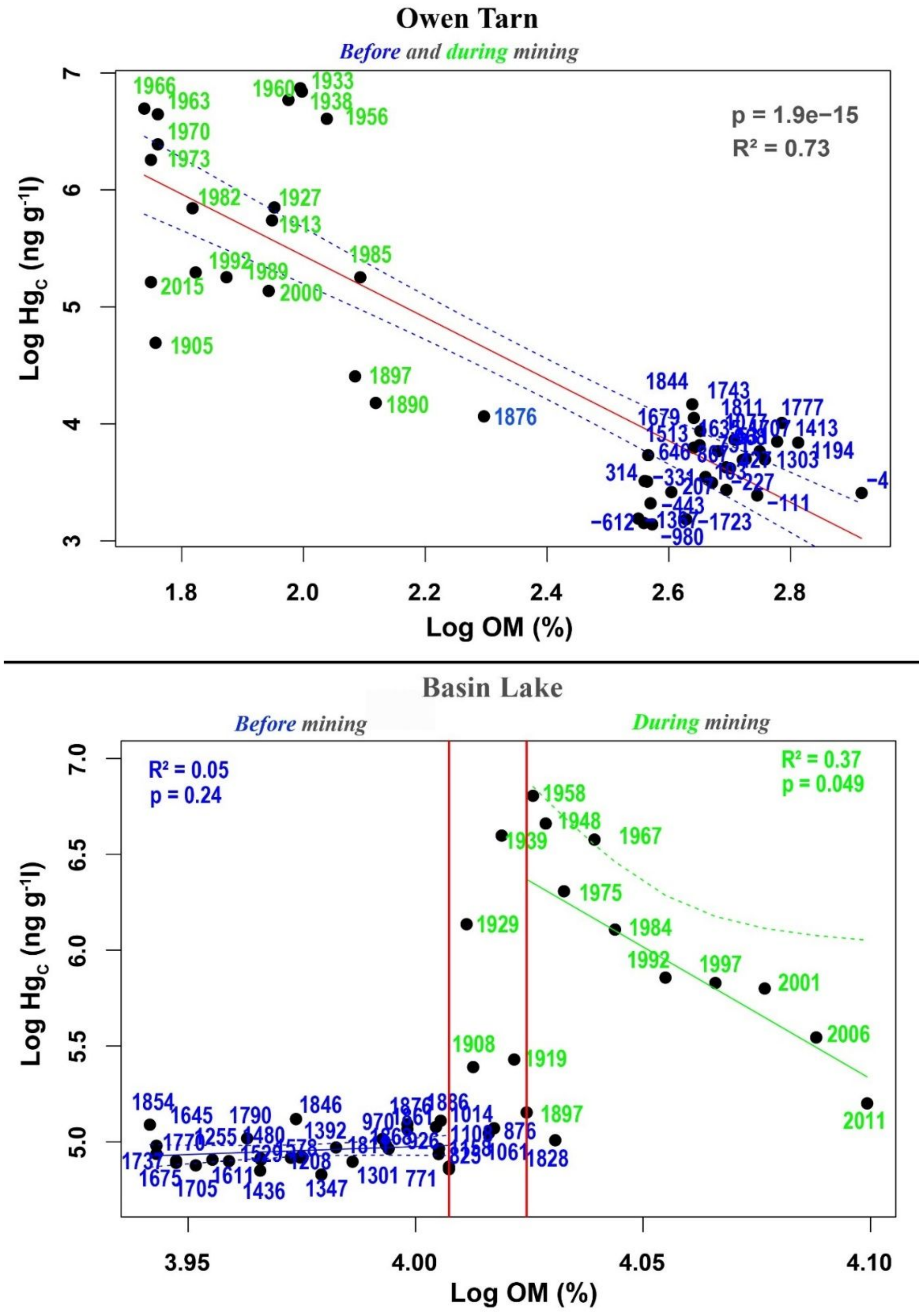

Supplementary Figure 9: Generalized Linear Model (GLM) of log-transformed mercury concentration (log Hg_C) and log-transformed organic matter concentration (log OM) in Owen Tarn (top) and Basin Lake (bottom) from Western Tasmania, Australia. For Basin Lake, a change point analysis for the change on mean and one for the change of variance were used to identify the transition zone and then two GLMs were created coincidently these change points show the change before and during mining.

Supplementary References

(1) Connor, S. E.; Schneider, L.; Trezise, J.; Rule, S.; Barrett, R. L.; Zawadzki, A.; Haberle, S. G. Forgotten Impacts of European Land-Use on Riparian and Savanna Vegetation in Northwest Australia. *J. Veg. Sci.* **2018**, *29* (3), 427–437. https://doi.org/10.1111/jvs.12591.

(2) Dunlap, T. R. Australian Nature, European Culture: Anglo Settlers in Australia. *Environ. Hist. Rev.* **1993**, *17* (1), 25–48. https://doi.org/10.2307/3984889.

(3) Li, K.; Lin, B. Impacts of Urbanization and Industrialization on Energy Consumption/CO2 Emissions: Does the Level of Development Matter? *Renew. Sustain. Energy Rev.* **2015**, *52*, 1107–1122. https://doi.org/10.1016/j.rser.2015.07.185.

(4) Newell, E.; Watts, S. The Environmental Impact of Industrialisation in South Wales in the Nineteenth Century: "Copper Smoke" and the Llanelli Copper Company. *Environ. Hist.* **1996**, *2* (3), 309–336.

(5) Sun, R.; Hintelmann, H.; Wiklund, J. A.; Evans, M. S.; Muir, D.; Kirk, J. L. Mercury Isotope Variations in Lake Sediment Cores in Response to Direct Mercury Emissions from Non-Ferrous Metal Smelters and Legacy Mercury Remobilization. *Environ. Sci. Technol.* **2022**, *56* (12), 8266–8277. https://doi.org/10.1021/acs.est.2c02692.

(6) Blainey, G. *The Peaks of Lyell*; St David's Park: Hobart, Tas., Australia, 1993.

(7) Weston, T. Mining Lower Grade Ore: Changes in Mining Technology at Mount Lyell, Tasmania, 1927-1939. *J. Australas. Min. Hist.* **2010**, *3*, 172–183.

(8) Chemical Engineering and Mining Review. Mount Lyell Smelting Practice. **1941**, 103–106.

(9) Hylander, L. D.; Herbert, R. B. Global Emission and Production of Mercury during the Pyrometallurgical Extraction of Nonferrous Sulfide Ores. *Environ. Sci. Technol.* **2008**, *42* (16), 5971–5977. https://doi.org/10.1021/es800495g.

(10) Hylander, L. D.; Meili, M. 500 Years of Mercury Production: Global Annual Inventory by Region until 2000 and Associated Emissions. *Sci. Total Environ.* **2003**, *304* (1), 13–27. https://doi.org/10.1016/S0048-9697(02)00553-3.

(11) Walch, J. *The Mount Lyell Mining and Railway Company Limited*; J. Walch & Sons: Hobart, Tasmania. Australia, 1946.

(12) Mudd, G. M. An Analysis of Historic Production Trends in Australian Base Metal Mining. *Ore Geol. Rev.* **2007**, *32* (1), 227–261. https://doi.org/10.1016/j.oregeorev.2006.05.005.

(13) The Mercury. Mt Luell Mines: Increased Efficiency of Flotation Plant. *1921*. Hobart, Tasmania. Australia June 23, 1922, p 2.

(14) McQuade, C. V.; Johnston, J.; Innes, S. *Review of Historical Literature and Data on the Sources and Quality of Effluent from the Mount Lyell Lease Site.*; Mount Lyell Remediation Research and Demonstration Program; 104; Comonwealth Department of Environment: Canberra, ACT. Australia., 1995.

(15) Corbett, K. D.; Solomon, M. Cambrian Mt Read Volcanics and Associated Mineral Deposits. In *Geology and Mineral Resources of Tasmania*; Burrett, C. F., Martin, E. L., Eds.; Special Publication; Geologial Society of Australia: Hobart, Tasmania, 1989; pp 84–153.

(16) Hodgson, D. A.; Vyverman, W.; Chepstow-Lusty, A.; Tyler, P. A. From Rainforest to Wasteland in 100 Years: The Limnological Legacy of the Queenstown Mines, Western Tasmania. *Fundam. Appl. Limnol.* **2000**, *149* (1), 153–176. https://doi.org/10.1127/archiv-hydrobiol/149/2000/153.

(17) Langford, J. Weather and Climate. In *Atlas of Tasmania*; Davies, J., Ed.; Lands and Surveys Department: Hobart, Tasmania. Australia, 1965; p 128.

(18) Harrison, J.; Heijnis, H.; Caprarelli, G. Historical Pollution Variability from Abandoned Mine Sites, Greater Blue Mountains World Heritage Area, New South Wales, Australia. *Environ. Geol.* **2003**, *43* (6), 680–687. https://doi.org/10.1007/s00254-002-0687-8.
(19) Schneider, L.; Pain, C. F.; Haberle, S.; Blong, R.; Alloway, B. V.; Fallon, S. J.; Hope, G.; Zawadzki, A.; Heijnis, H. Evaluating the Radiocarbon Reservoir Effect in Lake Kutubu, Papua New Guinea. *Radiocarbon* **2018**, 1–22. https://doi.org/10.1017/RDC.2018.49.
(20) Blaauw, M.; Christen [aut, J. A.; Lopez, M. A. A.; Vazquez, J. E.; Gonzalez, O. M.; Belding, T.; Theiler, J.; Gough, B.; Karney, C. Rplum: Bayesian Age-Depth Modelling of Cores Dated by Pb-210, 2024. https://cran.r-project.org/web/packages/rplum/index.html (accessed 2025-02-19).
(21) Hogg, A. G.; Heaton, T. J.; Hua, Q.; Palmer, J. G.; Turney, C. S.; Southon, J.; Bayliss, A.; Blackwell, P. G.; Boswijk, G.; Ramsey, C. B.; Pearson, C.; Petchey, F.; Reimer, P.; Reimer, R.; Wacker, L. SHCal20 Southern Hemisphere Calibration, 0–55,000 Years Cal BP. *Radiocarbon* **2020**, *62* (4), 759–778. https://doi.org/10.1017/RDC.2020.59.
(22) Wang, Q.; Li, Y.; Wang, Y. Optimizing the Weight Loss-on-Ignition Methodology to Quantify Organic and Carbonate Carbon of Sediments from Diverse Sources. *Environ. Monit. Assess.* **2011**, *174* (1–4), 241–257. https://doi.org/10.1007/s10661-010-1454-z.
(23) Foucher, D.; Hintelmann, H. High-Precision Measurement of Mercury Isotope Ratios in Sediments Using Cold-Vapor Generation Multi-Collector Inductively Coupled Plasma Mass Spectrometry. *Anal. Bioanal. Chem.* **2006**, *384* (7), 1470–1478. https://doi.org/10.1007/s00216-006-0373-x.
(24) Ma, J.; Hintelmann, H.; Kirk, J. L.; Muir, D. C. G. Mercury Concentrations and Mercury Isotope Composition in Lake Sediment Cores from the Vicinity of a Metal Smelting Facility in Flin Flon, Manitoba. *Chem. Geol.* **2013**, *336*, 96–102. https://doi.org/10.1016/j.chemgeo.2012.10.037.
(25) Stein, A. F.; Draxler, R. R.; Rolph, G. D.; Stunder, B. J. B.; Cohen, M. D.; Ngan, F. NOAA's HYSPLIT Atmospheric Transport and Dispersion Modeling System. *Bull. Am. Meteorol. Soc.* **2015**, *96* (12), 2059–2077. https://doi.org/10.1175/BAMS-D-14-00110.1.
(26) Draxler, R. R.; Hess, G. D. An Overview of the HYSPLIT 4 Modelling System for Trajectories, Dispersion and Deposition. *Aust. Meteorol. Mag.* **1998**, *47*, 295–308.
(27) Carslaw, D. C.; Ropkins, K. *Openair* — An R Package for Air Quality Data Analysis. *Environ. Model. Softw.* **2012**, *27–28*, 52–61. https://doi.org/10.1016/j.envsoft.2011.09.008.
(28) Goyal, R.; Sen Gupta, A.; Jucker, M.; England, M. H. Historical and Projected Changes in the Southern Hemisphere Surface Westerlies. *Geophys. Res. Lett.* **2021**, *48* (4), e2020GL090849. https://doi.org/10.1029/2020GL090849.
(29) ANZG. *Australian and New Zealand Guidelines for Fresh and Marine Water Quality, Australian and New Zealand Governments and Australian state and territory governments, Canberra ACT, Australia.* www.waterquality.gov.au/anz-guidelines.
(30) Lintern, A.; Schneider, L.; Beck, K.; Mariani, M.; Fletcher, M.-S.; Gell, P.; Haberle, S. Background Concentrations of Mercury in Australian Freshwater Sediments: The Effect of Catchment Characteristics on Mercury Deposition. *Elem. Sci. Anthr.* **2020**, *8* (1), 019. https://doi.org/10.1525/elementa.019.
(31) Killick, R.; Eckley, I. A. Changepoint: An R Package for Changepoint Analysis. *J. Stat. Softw.* **2014**, *58*, 1–19. https://doi.org/10.18637/jss.v058.i03.
(32) Harle, K. J.; Britton, K.; Heijnis, H.; Zawadzki, A.; Jenkinson, A. V. Mud, Mines and Rainforest: A Short History of Human Impact in Western Tasmania, Using Pollen, Trace Metals and Lead-210. *Aust. J. Bot.* **2002**, *50* (4), 481–497. https://doi.org/10.1071/bt01028.

(33) Gao, X.; Yuan, W.; Chen, J.; Huang, F.; Wang, Z.; Gong, Y.; Zhang, Y.; Liu, Y.; Zhang, T.; Zheng, W. Tracing the Source and Transport of Hg during Pedogenesis in Strongly Weathered Tropical Soil Using Hg Isotopes. *Geochim. Cosmochim. Acta* **2023**, *361*, 101–112. https://doi.org/10.1016/j.gca.2023.10.009.
(34) Zhang, H.; Fu, X.; Wu, X.; Deng, Q.; Tang, K.; Zhang, L.; Sommar, J.; Sun, G.; Feng, X. Using Mercury Stable Isotopes to Quantify Bidirectional Water–Atmosphere Hg(0) Exchange Fluxes and Explore Controlling Factors. *Environ. Sci. Technol.* **2023**, *57* (29), 10673–10685. https://doi.org/10.1021/acs.est.3c01273.
(35) Sun, R.; Streets, D. G.; Horowitz, H. M.; Amos, H. M.; Liu, G.; Perrot, V.; Toutain, J.-P.; Hintelmann, H.; Sunderland, E. M.; Sonke, J. E. Historical (1850–2010) Mercury Stable Isotope Inventory from Anthropogenic Sources to the Atmosphere. *Elem. Sci. Anthr.* **2016**, *4*, 000091. https://doi.org/10.12952/journal.elementa.000091.
(36) Meng, M.; Sun, R.; Liu, H.; Yu, B.; Yin, Y.; Hu, L.; Shi, J.; Jiang, G. An Integrated Model for Input and Migration of Mercury in Chinese Coastal Sediments. *Environ. Sci. Technol.* **2019**, *53* (5), 2460–2471. https://doi.org/10.1021/acs.est.8b06329.
(37) Carignan, J.; Estrade, N.; Sonke, J. E.; Donard, O. F. X. Odd Isotope Deficits in Atmospheric Hg Measured in Lichens. *Environ. Sci. Technol.* **2009**, *43* (15), 5660–5664.
(38) Sonke, J. E. A Global Model of Mass Independent Mercury Stable Isotope Fractionation. *Geochim. Cosmochim. Acta* **2011**, *75* (16), 4577–4590. https://doi.org/10.1016/j.gca.2011.05.027.
(39) Sun, R.; Hintelmann, H.; Wiklund, J. A.; Evans, M. S.; Muir, D.; Kirk, J. L. Mercury Isotope Variations in Lake Sediment Cores in Response to Direct Mercury Emissions from Non-Ferrous Metal Smelters and Legacy Mercury Remobilization. *Environ. Sci. Technol.* **2022**, *56* (12), 8266–8277. https://doi.org/10.1021/acs.est.2c02692.
(40) Sun, R.; Yuan, J.; Sonke, J. E.; Zhang, Y.; Zhang, T.; Zheng, W.; Chen, S.; Meng, M.; Chen, J.; Liu, Y.; Peng, X.; Liu, C. Methylmercury Produced in Upper Oceans Accumulates in Deep Mariana Trench Fauna. *Nat. Commun.* **2020**, *11* (1), 3389. https://doi.org/10.1038/s41467-020-17045-3.
(41) Denwer, K. P. Alteration and Mineral Zonation at the Mt Lyell Copper–Gold Deposit, Tasmania. *Aust. J. Earth Sci.* **2018**, *65* (6), 787–807. https://doi.org/10.1080/08120099.2018.1472663.
(42) Champion, D.; Raymond, O. L.; Huston, D. L.; VanDerWielen, S.; Sexton, M.; Bastreakov, E.; Schroder, I.; Butcher, G.; Hawkins, S. J.; Lane, J.; McAlpine, S.; Czarnota, K.; Britt, A.; Granitto, M.; Hofstra, A.; Kreiner, D.; Emsbo, P.; Kelley, K.; Wang, B.; Case, G.; Graham, G.; Lauzière, K.; Lawley, C.; Gadd, M.; Pilote, J.-L.; Létourneau, F.; Haji, E. A. *Critical Minerals in Ores - geochemistry database*. https://ecat.ga.gov.au/geonetwork/srv/eng/catalog.search#/metadata/145496 (accessed 2025-02-12).
(43) Caritat, P.; Reimann, C.; Chekushin, V.; Bogatyrev, I.; Niskavaara, H.; Braun, J. Mass Balance between Emission and Deposition of Airborne Contaminants. **1997**, *31* (10), 2966–2972. https://doi.org/10.1021/es970193z.
